# Mitochondrial Genome Polymorphisms in the Human Pathogenic Fungus *Cryptococcus neoformans*

**DOI:** 10.3389/fmicb.2020.00706

**Published:** 2020-04-21

**Authors:** Yue Wang, Jianping Xu

**Affiliations:** ^1^Department of Biology, McMaster University, Hamilton, ON, Canada; ^2^Institute of Bast Fiber Crops and Center of Southern Economic Crops, Chinese Academy of Agricultural Sciences, Changsha, China

**Keywords:** *Cryptococcus*, mtDNA inheritance, intron distribution, phylogeny, mating type, recombination

## Abstract

The *Cryptococcus* complex consists of at least seven evolutionary divergent lineages and causes ∼200,000 fatal human infections each year worldwide. The dominant lineage is *Cryptococcus neoformans* which consists of three haploid clades VNI, VNII, and VNB, their haploid hybrids, and various diploids derived from intra- and inter-clade mating events. In this study, we analyzed the mitogenomes of 184 strains of *C. neoformans*. Our analyses revealed that all 184 mitogenomes contained the same 15 protein-coding genes in the same gene order. However, their mitogenome sizes varied between 24,740 and 31,327 bp, primarily due to differences in the number and size of mitochondrial introns. Twelve nucleotide sites within five mitochondrial genes were found to contain introns in at least one of the 184 strains, ranging from 2 to 7 introns within each mitogenome. The concatenated mitochondrial exon sequences of the 15 protein-coding genes and two rRNA genes showed that VNI, VNII, and VNB strains were separated into distinct clades or sub-clades, largely consistent with results based on nuclear genome SNPs. However, several novel findings were observed. First, one strain of the VNB clade contained mitogenome exon sequences identical to the main VNI mitogenome type but was distant to other VNB mitogenomes. Second, hybrids among clades VNI, VNII, and VNB identified based on their nuclear genome SNPs contained mitogenomes from different clades, with evidence of their mitogenomes inherited from either the *MAT***a** or the *MAT***α** parents. Third, the eight diploid VNB (*C. neoformans*) × VNIV (*C. deneoformans*) hybrids contained recombinant mitogenomes. Fourth, analyses of intron distribution and the paired exon–intron phylogenies for each of the 12 exon–intron pairs suggested frequent gains and losses of mitochondrial introns during the evolution of *C. neoformans*. The combined mitogenome exon-based phylogeny and intron distributions suggested that clades VNI, VNII and VNB could be further divided into sub-clades. Together, our results revealed a dynamic evolution of mitochondrial genomes in this important human fungal pathogen.

## Introduction

The human pathogenic *Cryptococcus* species complex contains at least seven evolutionary divergent lineages and their hybrids (Hagen et al., 2015, Hagen et al., 2017; Kwon-Chung et al., 2017; Samarasinghe and Xu, 2018). Together, they are responsible for over 200,000 deaths per year globally (Rajasingham et al., 2017). Cryptococcal infections can occur in multiple forms and at multiple body sites, including respiratory infections, skin lesions, and meningoencephalitis. The most common and most deadly form of cryptococcal infection is Cryptococcal meningitis, a leading cause of death among HIV/AIDS patients worldwide (Rajasingham et al., 2017). These yeasts are commonly found in soils, bird droppings, and tree barks (Litvintseva et al., 2011; Samarasinghe and Xu, 2018). Currently, based on one school of thought, the seven evolutionarily divergent lineages within the species complex should each be recognized as a distinct *Cryptococcus* species (Hagen et al., 2017). The most common of these seven species is *Cryptococcus neoformans*, which has also been variably called *Cryptococcus neoformans* serotype A, *C. neoformans* var. *grubii*, *C. neoformans* molecular types (clades) VNI, VNII, and VNB in the literature (Hagen et al., 2017; Kwon-Chung et al., 2017; Samarasinghe and Xu, 2018). The focus of this paper is on this group of yeasts and we will use the species name *C. neoformans* to represent this group.

As a medically important organism, a large body of research has been conducted on *C. neoformans*, including establishing a diversity of animal models, identifying the genes associated with virulence, and investigating its molecular epidemiology and population genetics. Indeed, many types of molecular markers such as isozyme electrophoresis, PCR-fingerprinting, amplified fragment length polymorphisms (AFLP), PCR-restriction fragment length polymorphisms (RFLP), multilocus sequence typing (MLST), and whole-genome shotgun sequencing have been used to analyze the patterns of genetic variation from local to global scales for *C. neoformans* (Xu et al., 2000b; Cogliati, 2013; Rhodes et al., 2017). These studies have identified that *C. neoformans* is composed of three divergent haploid clades VNI, VNII, VNB, as well as hybrids among the three clades (Rhodes et al., 2017). In addition, sub-Sahara Africa has been shown to contain the highest genetic diversity and is most likely the center of origin for *C. neoformans* (Litvintseva et al., 2007). These studies have also revealed both ancient and recent migrations among regions within continents and between continents for strains in the different clades (Xu et al., 2000b; Cogliati, 2013; Rhodes et al., 2017). However, most of the analyses so far have focused on nuclear genes and genomes. There is very limited information on the variations of mitochondrial genes and genomes at the population level in *C. neoformans*.

The mitochondrion is an important organelle within eukaryotic cells, responsible for the production of ATP, the energy currency in living organisms. Aside from ATP generation, mitochondria are also involved in several other cellular processes, such as cell senescence and the maintenance of ion homeostasis (Burger et al., 2003; Chatre and Ricchetti, 2014). Within cells, mitochondria interact with multiple other membrane-bound structures including the nucleus, lysosomes, and the endoplasmic reticulum to impact cell survival and reproduction (Chatre and Ricchetti, 2014). In pathogenic fungi, mitochondria have been found to playing a role in virulence, regulating biofilm and hyphal growth, and activating drug resistance (Burger et al., 2003; Chatre and Ricchetti, 2014; Calderone et al., 2015). The mitochondrial genome in the type strain of *C. neoformans*, H99, has been sequenced and annotated (GenBank accession number NC004336). It has a genome size of 24,874 bp and contains 14 common fungal mitochondrial protein-coding genes associated with the electron transport chain and ATP synthesis (*ND1, ND2, ND3, ND4, ND4L, ND5, ND6, ATP6, ATP8, ATP9, COX1, COX2, COX3*, and *COB*). In addition, it contains the ribosomal protein S3 (*rps3*) and the small and large subunits of the mitochondrial ribosomal RNA (SsRNA and LsRNA) genes. Two other genes were also identified in the H99 mitogenome: ribonuclease P (*rnp*) and a hypothetical protein orf267. There are 20 tRNA genes in the H99 mitogenome, encoding 19 of the 20 amino acids (the tRNA-Cys is missing while there are two copies of the tRNA-Met gene). Two of the genes in the H99 mitogenome contain introns, a 1121 bp intron in the *ND1* gene and a 1142 bp intron in the *COB* gene. The two introns contain one open reading frame each coding for a protein with the LAGLIDADG motifs that are characteristic of homing endonuclease genes. The H99 mitogenome is over 20% smaller than that of JEC21 (33,194 bp), a model strain of a closely related species *Cryptococcus deneoformans* (Litter et al., 2005). The major contributor to the mitogenome size difference between H99 and JEC21 is the large number of introns in the JEC21 mitogenome.

*Cryptococcus neoformans* is predominantly a haploid. However, diploid and aneuploid strains can also be found in both clinical and environmental sources. It can reproduce both asexually via budding and sexually via mating (Zhao et al., 2019). During asexual reproduction, both the nuclear and mitochondrial genomes are faithfully transmitted from the parental cells to offspring. However, new mutations could emerge during asexual reproduction, including nucleotide substitutions and changes in chromosome structure and chromosome number, especially under certain selection pressure such as exposure to antifungal drugs (e.g., Hua et al., 2019; Dong et al., 2020). If asexual reproduction were the only mode of reproduction for *C. neoformans* in nature, we should observe strain relationships inferred based on their nuclear genome sequences to be similar to those based on their mitochondrial genome sequences. On the other hand, if sexual mating were common, due to their different inheritance patterns in sexual crosses (Xu et al., 2000a), strains relationships inferred based on their nuclear and mitochondrial genome sequences could be different.

Sexual mating in *C. neoformans* is controlled by one locus with two alternative alleles, *MAT***a** and *MAT***α**. In laboratory crosses involving *MAT***a** and *MAT***α** strains, the progeny primarily inherits mitochondria from the *MAT***a** parent (Yan and Xu, 2003). If sexual reproduction were common, most strains would show mixed ancestries in their nuclear genomes while their mitogenomes would follow those of their *MAT***a** parent. Consequently, at the population level, we would see linkage equilibrium among alleles at different nuclear loci but mitogenome markers may be different. Population genetic and genomic studies based on nuclear genome markers have revealed evidence for both sexual and asexual reproductions in natural populations of *C. neoformans* (Hiremath et al., 2008; Rhodes et al., 2017). However, same-sex mating between strains of *MAT*α is also possible and in such a cross, as shown in laboratory crosses, biparental mitochondrial inheritance, including recombinant mitogenomes, may be observed among progeny (Yan et al., 2004).

The objectives of this study are to analyze the patterns of variation of mitochondrial genes and genomes in natural populations of *C. neoformans*. We focus on analyzing the mitochondrial genome data obtained and released in Rhodes et al. (2017). In the Rhodes et al. (2017) study, they sequenced the genomes of 188 *C. neoformans* strains and analyzed single nucleotide polymorphisms and copy number variations for nuclear genes in these strains. However, their mitochondrial genomes were not analyzed. Thus, the significance of the mitogenome in the evolution of *C. neoformans* remains unknown. The 188 strains that they sequenced were selected to represent *C. neoformans* from different geographic regions, different ecological niches, and/or different MLSTs at seven nuclear loci. However, the sample sizes representing different geographic regions, ecological niches, and MLST were not balanced (Rhodes et al., 2017; Supplementary Tables S1, S2). In this study, we extracted the mitochondrial DNA sequences from each of those strains, assembled their mitochondrial genomes, and analyzed the patterns of genome size variation, intron distribution variation, and single nucleotide polymorphisms. Our analyses identified several novel insights in the evolution of this important human fungal pathogen.

## Materials and Methods

### Strains and Nuclear Genotype Information

The strains analyzed here were from the study by Rhodes et al. (2017, see their Supplementary Table S1 for details). Briefly, these strains came from 14 different countries representing all major regions of the world where *C. neoformans* have been found (Africa, Asia, Australia, Caribbean, Europe, and North and South America). These strains were isolated from different ecological niches (bird guano, soil, trees, animals, and humans). In addition, they were selected for genome sequencing based on their MLST results at seven nuclear gene fragments so as to be representative of the genetic diversity in their respective geographic regions. Thus, even though this collection of strains may not represent the true global population structure of *C. neoformans*, they likely capture most of the global genetic diversity of this species. As a result, we believe that the mitogenomes from these strains should similarly represent the mitogenome diversity of the global *C. neoformans*.

**FIGURE 1:**
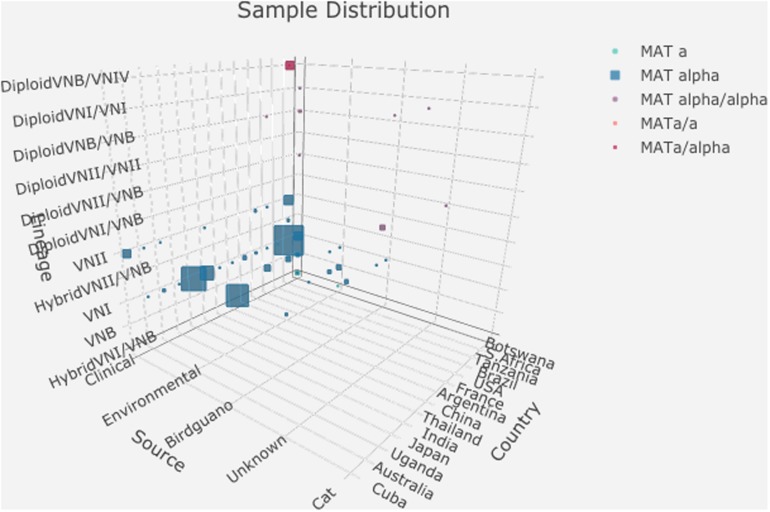
Distributions of 188 strains based on their geographic origin, ecological niche, mating type, and nuclear ploidy, and clade. The detailed data used to generate this graph is presented in Supplementary Table S2.

Analyses of the nuclear genomes from the 188 strains revealed that 157 of the 188 isolates were haploid and belonged to one of three major clades (VNI, VNII, and VNB), consistent with results from previous MLST studies of these strains. However, seven haploid isolates showed hybrid ancestry, including five VNI/VNB hybrids and two VNII/VNB hybrids. The remaining 24 isolates displayed evidence of heterozygosity within individual strains, consistent with diploidy or aneuploidy. Genomic analyses revealed that these 24 diploid isolates were likely the mating products of: (i) between strains from within the same lineage (VNI × VNI, 4 strains; VNII × VNII, 4 strains; and VNB × VNB, 3 strains); (ii) between different lineages within *C. neoformans* (VNI × VNB, 2 strains; VNII × VNB, 2 strains); (iii) between two species *C. neoformans* × *C. deneoformans* (8 strains); and (iv) between *C. neoformans* × *Cryptococcus gattii* (1 strain) (Rhodes et al., 2017). The distributions of these 188 strains based on their geographic, ecological, mating type, and nuclear genotypes are summarized in Supplementary Tables S1, S2. Figure 1 shows a simplified representation of sample distributions in a 3D bubble plot generated using the R package ‘plotly 4.9.1’^1^. Among the 188 strains analyzed in this study, 112 were the VNI molecular type (60%), 158 were *MAT***α** (84%), and 141 were from human patients (75%) (Supplementary Tables S1, S2). Thus, other molecular types (VNII, VNB, and their hybrids), *MAT***a** strains, and non-clinical samples such as those from the environment were under-represented.

### Mitogenome Sequence Data Retrieval and Assembly

For all 188 strains, their whole-genome sequence data were retrieved from NCBI database according to the Sequence Read Archive (SRA) accession numbers provided by Rhodes et al. (2017, Supplementary Table S1 in their study). Since all the sequences were generated by paired-end sequencing, fastq-dump from sratoolkit (Leinonen et al., 2010) was used to extract fastq files from the run files and separated them into forward and reverse files.

The program NOVOPlasty3.7 (Dierckxsens et al., 2017) was used to assemble the mitochondrial genome for each of the strains. For each configuration file, the project name, forward read path, and reverse read path were specified. The initial genome size range was set as 19000–35000 bp which covers the known mitogenome size range for all varieties of the human pathogenic *Cryptococcus* species complex (Litter et al., 2005). The *ND5* gene sequence from isolate H99 was used as the seed sequence to retrieve the overlapping reads from the fastq files and then initiate the assembly, using NOVOPlasty3.7. The whole mitogenome sequence of H99 was given as a reference to help resolve duplicated regions in each mitogenome when necessary. Assembly parameters used those in the default setting as recommended.

### Mitogenome Annotation

The fungal mitogenomes vary significantly in their genome size and gene content (Sandor et al., 2018). At present, there is no analytical pipeline developed specifically for annotating fungal mitogenomes. We tried two programs for our annotation: MitoS (Bernt et al., 2013) and MitoZ (Meng et al., 2019). MitoS is a popular webserver that requires assembled mitogenomes as input files for annotation and it annotates one mitogenome at a time. In contrast, MitoZ is a pipeline consisting of several independent modules of *de novo* assembly, findMitoScaf (find Mitochondrial Scaffolds), annotation and visualization. MitoZ was originally developed to assemble and annotate animal mitogenomes (which are generally conserved in their size and genome content) (Meng et al., 2019). However, it can generate mitogenome assembly together with annotation and visualization results from high throughput raw sequence reads. For protein coding gene annotation, MitoZ allows user-defined Perl script to combine with BLAST and user-defined python script to precisely locate the start and stop codons through batch processing. In this study, we modified the protocols of the program MitoZ_v2.44 to annotate the 188 *C. neoformans* mitogenomes. Because the published Cryptococcal mitogenomes are known to be larger and contain more genes than animal mitogenomes, we replaced the list of animal mitochondrial genes with known Cryptococcal mitochondrial genes already published and deposited in GenBank, including building new HMM profiles for all the Cryptococcal genes. To build the HMM profiles, we first downloaded all the known mitochondrial gene sequences of the *Cryptococcus* species complex from the NCBI database. We then aligned the multiple sequences for each gene using MAFFT v7.429 (Katoh et al., 2002). The alignment for each *Cryptococcus* mitochondrial gene was then imported into HMMER_v3.2.1^2^ to build the HMM profiles. Since most animal mitochondrial genomes do not contain introns, the program MitoZ does not implement the HMM model to identify introns. Thus, after assembly and annotation through MitoZ, we used a greedy BLAST to separate exons and intron(s) within each gene (Zhang et al., 2000).

### Analyses of Mitogenome Size Distributions

The distributions of mitogenome sizes among the assembled mitogenomes were analyzed using Boxplot to compare strains belonging to different categories such as geographic origins, ecological niches, mating types, and genetic lineages. The statistical significance of the differences in mitogenome sizes and intron numbers among groups within each category was determined based on one-way ANOVA. The distributions of mitogenome size and intron number with different sample characteristics were drawn with R package ‘ggplot2’ (Wickham, 2016). In these graphs, to help visualization of the number of data points around each intron number category and each mitogenome size category, we artificially introduced 0.2 intron as the standard deviation around each intron number category and 200 bp as the standard variation around each mitogenome size category.

### Phylogenetic Analyses

Phylogenetic analyses were conducted with package phangorn 2.5.5 (Schliep, 2011) in R. Two types of phylogenetic analyses were performed. In the first, we concatenated the exon sequences of all 17 known and shared coding genes (*ND1, ND2, ND3, ND4, ND4L, ND5, ND6, ATP6, ATP8, ATP9, COX1, COX2, COX3, COB, RPS3, LsRNA, and SsRNA*) in the mitogenomes and aligned them with msa:msaMuscle (Bodenhofer et al., 2015). A Neighbor-Joining (Saitou and Nei, 1987) tree based on the mean nucleotide sequence differences between strains was built. Statistical supports for individual branches were assessed based on 1000 bootstraps. The relevant strain features such as geographic origins, ecological niches, mating type, lineage/genotype group, and intron distributions were plotted along the concatenated exon phylogenetic tree using the program ggtree 1.14.6 (Yu et al., 2017).

The second type of phylogenetic analyses was an individualized exon–intron phylogeny comparison for each intron and its corresponding exon sequences. In our *C. neoformans* mitogenome assemblies, each of 12 nucleotide sites within five genes contained an intron from multiple strains (see section “Results” below). These five genes were (i) the large subunit of the ribosomal RNA (*LsRNA*) gene (four positions within this gene contained introns); (ii) *COB* (two positions within this gene contained introns); (iii) *COX1* (four positions within this gene contained introns); (iv) *COX2* (one position within this gene contained intron); and (v) *ND1* (one position within this gene contained intron). For introns at each of these 12 positions, we first aligned the intron sequences and obtained a phylogeny using the NJ method in MEGA X (Kumar et al., 2018). Similarly, the exon phylogeny for each of the five genes was also constructed. These paired exon and intron phylogenies were then compared with each other. The exon–intron co-phylogenies were constructed using R ‘phytools 0.7-02’ (Revell, 2012).

## Results

We successfully extracted and assembled the mitochondrial genome sequences of 183 strains, each into a single circularized molecule. Of the remaining five strains, we were unable to assemble a circularized mitogenome. These five strains were not included into the subsequent analyses. However, we included the already assembled H99 mitogenome into our analyses as a reference, giving a total of 184 assembled *C. neoformans* mitogenomes for our comparative analyses. Among the 184 assembled mitogenomes, their genome sizes varied between 24,740 and 31,327 bp long (Supplementary Table S1). All assembled mitogenomes contained the same set of 17 genes in the same following order: *ND2, ND3, COX2, ND1, COB, SRP3, LsRNA, COX3, ND4, ND4L, ND5, ND6, SsRNA, ATP6, ATP9, COX1*, and *ATP8.* This gene order is also identical to those of the mitogenomes in strain B3501A of the closely related species *C. deneoformans* and in strain R265 of *Cryptococcus gattii* (Litter et al., 2005; Ma and May, 2010). All 184 mitogenomes contained introns and their intron numbers varied between 2 and 7. These introns were distributed across 12 different nucleotide sites located within five genes *LsRNA, COB, COX1, COX2*, and *ND1*. Below we first describe the main results with regard to mitogenome size and intron distributions among clades/genotype groups, geographic populations, ecological niches, and mating types. This is then followed by results from the analyses of nucleotide polymorphisms in either the exons and/or introns in these mitogenomes.

### Mitogenome Size and Intron Number Distributions

#### Distributions Based on Clades/Nuclear Genotypes

**FIGURE 2:**
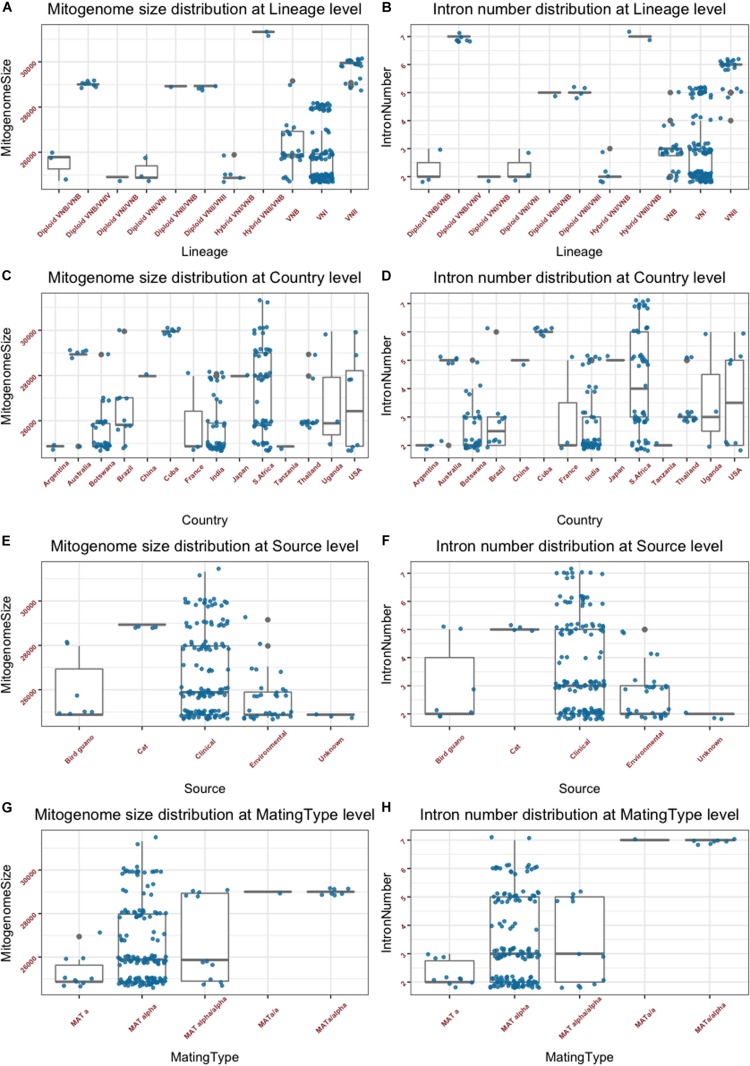
Mitogenome sizes and intron numbers distribution among strain features as defined based on various characteristics. **(A,B)** Variations within and among clades and genotype groups as defined based on nuclear genome SNPs. **(C,D)** variations within and among their geographic (country) origins. **(E,F)** Variations within and among ecological niches. **(G,H)** Variations within and among different mating types or mating type combinations. Random noise was added to plots using a uniform distribution with 0.38 horizontal jitter. The vertical jitter was set as 200 bp for genome size and 0.2 for intron number.

The summary mitogenome size and intron number distributions within and among clades/genotype groups are presented in Figures 2A,B. Among the three haploid clades VNI, VNII, and VNB, the mean mitogenome size of VNII is significantly bigger than those of VNI and VNB (*p* < 0.0001 in both cases), but those of VNI and VNB not significantly different from each other (*p* = 0.49). Of the remaining genotype groups, the two VNII/VNB haploid hybrids had the biggest mitogenome sizes while those of the diploid VNI/VNB hybrids, haploid VNI/VNB hybrids, and diploid VNI/VNI strains had among the smallest mitogenomes (Figure 2A). However, due to the limited sample sizes in these genotype categories, statistical tests in the significance of their mitogenome size differences were not determined. The intron numbers showed an overall distribution parallel to that of the mitogenome size distributions (Figures 2A,B). We note, however, that there are some subtle differences between the two. For example, the eight diploid VNB/VNIV hybrids all had seven introns in each of its mitogenome, the highest intron number in a given mitogenome among the 184 strains but their mitogenome sizes were not the biggest (Figures 2A,B and Supplementary Table S1).

#### Distribution Based on Geographic Origins

The summary mitogenome size and intron number distributions within and among geographic regions (countries) are presented in Figures 2C,D. Most countries with sample sizes relatively large showed a range of mitogenome sizes (Figure 2C and Supplementary Table S1). The only exception seemed to be the seven strains from Cuba that all had an identical mitogenome size of 29,951 bp. We conducted one-way pairwise ANOVA for the three geographic populations with large sample sizes: Botswana, India, and South Africa. Our analyses showed that overall, the South African population had an average mitogenome size significantly larger than those from Botswana and India (*p* < 0.0001 in both cases) while no significant size difference was found between those from Botswana and India (*p* = 0.82). Again, the intron number distributions within and between the different countries were similar to those of mitogenome size distribution (Figures 2C,D).

#### Distribution Based on Ecological Niches

The summary mitogenome size and intron number distributions within and among ecological niches are presented in Figures 2E,F. Most of the 184 strains were from clinical sources, with relatively few from other sources. Of the four types of ecological niches as classified in the Rhodes et al. (2017) study, three showed a range of mitogenome size and intron number variations among strains (Figures 2E,F). One-way ANOVA results show that the clinical population of *C. neoformans* from humans had an average mitogenome size significantly larger than that from environmental sources [*p* = 0.002; for this analysis, due to small sample size for the bird-guano population, we combined the bird-guano strains with the “environmental strains” in Rhodes et al. (2017) into one “environmental sample.”]. Interestingly, all four strains from cats were diploid VNII/VNII isolates from Australia and they had the same mitogenome size (28,932 bp), contained the same number of mitochondrial introns (5) at exactly the same mitogenome locations (Supplementary Table S2), and had identical mitogenome exon sequences (see section “Results” below).

#### Distributions Based on Mating Types

The summary mitogenome size and intron number distributions among mating type groups are presented in Figures 2G,H. For both mitogenome size and intron number, *MAT***α** strains showed greater ranges of variation than those of *MAT***a** strains. However, there was no statistically significant difference between the *MAT***a** and *MAT***α** populations in their mean mitogenome sizes. Interestingly, of the three remaining mating type combinations (*MAT***a/a**, *MAT***α/a**, and *MAT***α/α**), two (the *MAT***a/a** and *MAT***a/α**) of them showed similar mitogenome sizes and intron numbers to each other while the *MAT***α/α** combination showed a broader range for both mitogenome size and intron number than those of *MAT***a/a** and *MAT***a/α**. The eight strains of the *MAT***a/a** and *MAT***a/α** mating types were of the diploid VNB/VNIV hybrids from South Africa. Based on their nuclear genotypes, these eight strains were likely descendants of the same recent hybridization event. Indeed, they all shared the same mitogenome size and intron number (Supplementary Table S1).

**FIGURE 3:**
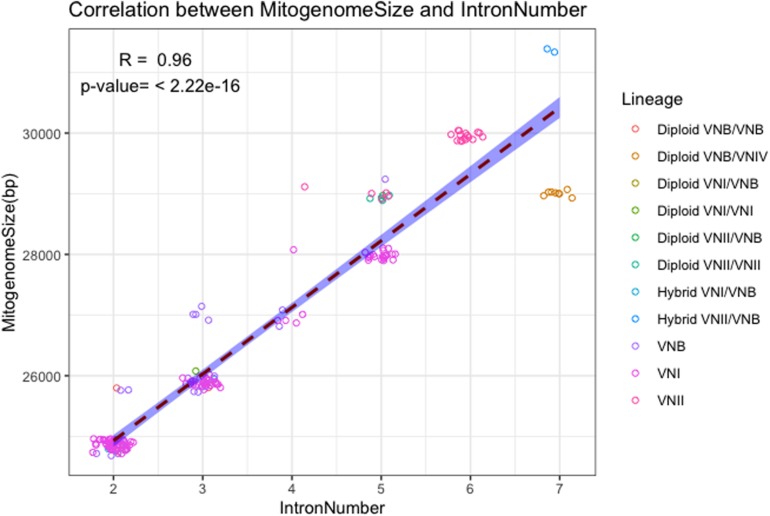
Correlation between mitogenome size and intron number. The regression line and the 95% confidence interval are shown. Random noise was added to points using normal distribution to show the number of data points around each value. The standard deviations given to the X axis (intron number) and the Y axis (mitogenome size) are 0.1 intron and 60 bp respectively.

The close association between mitogenome size and intron number as demonstrated in Figure 2 is further confirmed in the direct correlational analyses between them using the 184 strain. As shown in Figure 3, a statistically highly significant correlation, with a high correlation coefficient, was found between mitogenome size and intron number in the global population of *C. neoformans*. As expected, the length of summed intron sequences was significantly correlated with mitogenome size (*r* = 0.980, *p* = 4.41E-131) (Supplementary Table S2). Interestingly, the summed lengths of intergenic regions and the summed lengths of exon sequences were also positively correlated with mitogenome sizes, with correlation coefficients of 0.707 (*p* = 2.20E-29) and 0.241 (*p* = 9.61E-3) respectively (Supplementary Table S2).

#### Concatenated Exon Sequence Phylogeny

**FIGURE 4:**
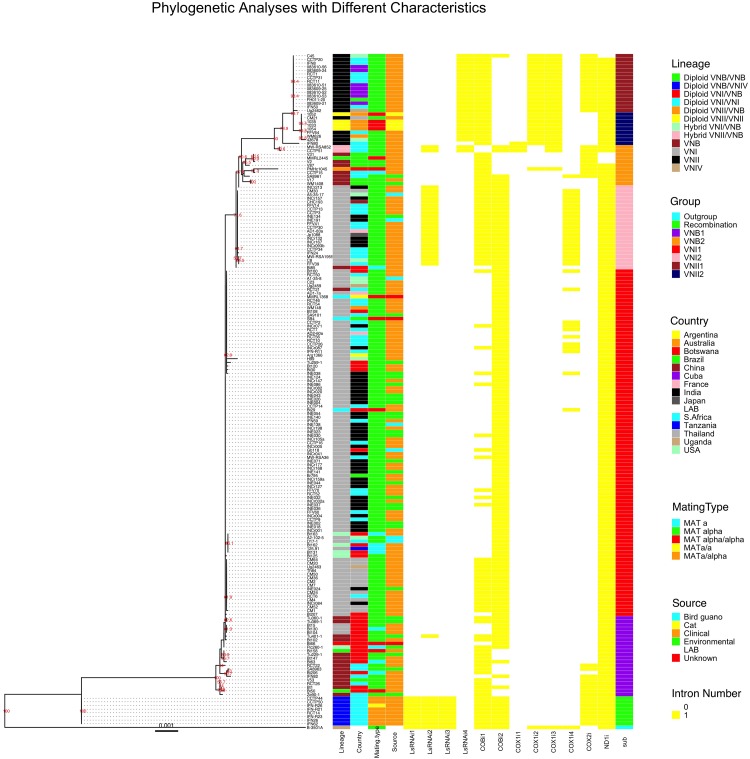
Mitochondrial concatenated exon phylogenetic tree of the 184 strains and the distributions of strains’ lineage, country of origin, mating type, ecological niche, each of the 12 introns, and sub-clade categorization. The maximum likelihood tree was constructed based on concatenated sequences of all exons in the mitochondrial genome. Different characteristics were then mapped to the tree.

We used the concatenated exon sequences from all 17 *C. neoformans* mitochondrial coding genes to construct a phylogeny (Figure 4). This phylogenetic tree was then used as a reference to show the phylogenetic distributions of all 184 strains based on their clade affiliations (as determined based on nuclear genome SNPs), ecological and geographic origins, and mating types (Rhodes et al., 2017). In addition, the distributions of individual introns were also mapped onto the mitochondrial exon phylogenetic tree (Figure 4). Below we provide brief descriptions of the mitogenome phylogeny and the distributions of various strain features.

#### Mitogenome Clades and Subclades

Our phylogenetic analysis separated the 184 mitogenomes into several large clades as well as a few intermediate types (Figure 4). Among the 184 strains, the most distantly related mitogenomes were from the eight diploid VNB/VNIV hybrids. This was then followed by one group of VNB strains (sub-clade VNB1, containing both haploid VNB and diploid VNB/VNB strains), strains of VNI (both haploid VNI and diploid VNI strains, in two distinct groups, sub-clades VNI1 and VNI2), the second group of VNB strains (sub-clade VNB2, containing both haploid VNB and diploid VNB/VNB strains), and finally the two sub-clades of VNII strains (sub-clade VNII1 contains both haploid VNII and diploid VNII strains while sub-clade VNII2 contains only haploid VNII strains) (Figure 4). The mitogenomes of hybrids (VNI/VNB, VNII/VNB hybrids) are dispersed within or between these groups and these will be described in detail in a latter section.

#### Main Differences Between Mitogenome Exon Phylogeny and Nuclear Genome SNP Phylogeny

Aside from the phylogenetic distinctions of mitogenomes within and among the main clades described above, we also note three major differences between the phylogeny based on the mitogenome exon sequences and that based on nuclear genome SNPs as shown in Rhodes et al. (2017). In the first, the mitogenomes of haploid VNB strains were clustered into two distinct sub-clades separated by a large cluster mostly composed of VNI strains (Figure 4). In addition, the mitogenome clustering patterns of the VNB strains were different from those based on nuclear genome SNPs. For example, strains V2, V31, V87, CCTP15, SA8961, V17, WM1408 were clustered together on the mitogenome exon phylogeny (in the VNB2 sub-clade). However, based on nuclear genome SNPs, while strains V2, V31 and V87 were clustered together, the other four strains were dispersed into a different cluster (Rhodes et al., 2017). Second, the VNI strains were separated into two phylogenetically distinct sub-clades VNI1 and VNI2, with each subclade having overall more similar mitogenome exon sequences to the VNB1 and VNB2 sub-clades respectively (Figure 4). Importantly, sub-clade VNI2 consists of 21 strains from diverse geographic areas including China (CHC193), India (6 strains such as INCr132), Thailand (CM30), the US (A5-35-17 and C8), South Africa (9 strains such as CCTP3), France (AD1-7a), and Japan (Jp1088). These 21 strains also formed a genotypic cluster based on nuclear genome SNPs as shown by Rhodes et al. (2017). Third, several VNB clade strains identified based on their nuclear genome SNPs showed very similar (e.g., strain Bt85 from Botswana) or identical (strain RCT21 from South Africa) mitogenome exon sequences to those of VNI1 and VNI2 sub-clades respectively (Figure 4). This result suggested that these strains were likely hybrids, with their mitochondrial and nuclear genomes within each strain belonging to different lineages.

#### Mitogenomes of Diploid Strains

The diploid VNI/VNI, VNB/VNB, VNII/VNII strains all have mitogenome exon sequences within the predicted main VNI, VNB, and VNII clades respectively. For example, all four VNII/VNII diploid strains have identical mitochondrial exon sequences to each other and clustered within the haploid VNII2 sub-clade. Similarly, of the three VNB/VNB diploid strains, one (MMRL2445) was clustered within the haploid VNB2 sub-clade while the other two (Bt50 and Bt158) were clustered within the VNB1 sub-clade. The three VNI/VNI diploid strains were all clustered within the main VNI1 sub-clade. However, the inter-clade diploid hybrids showed inconsistent mitogenome exon phylogenetic distributions. For example, the diploid VNI/VNB strain Bt66 has a mitogenome exon genotype clustered within one of the VNB sub-clades (Figure 4). In contrast, two diploid VNII/VNB hybrids (MW-RSA852 and CCTP51) had intermediate mitochondrial exon genotypes between VNB and VNII. For the eight diploid VNB/VNIV hybrids, they all showed mitogenomes very different from the known VNB mitogenomes as well as distinct from the reference mitogenome of VNIV (*C. deneoformans*) (Figure 4). These results suggest the potential biparental mitochondrial inheritance and/or mitogenome recombination during hybridization in nature.

#### Mitogenomes of Haploid Hybrids

Of the seven haploid hybrids among clades within *C. neoformans*, their mitogenome exon sequences also showed different phylogenetic distribution patterns. For example, of the five VNI/VNB haploid hybrids as identified based on their nuclear genome SNPs, four (Bt125, Bt131, Bt162, and Bt163) had mitogenomes more similar to those of the main VNI1 sub-clade while one strain (Ftc260-1) was clustered into the VNB1 sub-clade (Figure 4).

#### Geographic Distributions on Mitogenome Tree

With regard to geographic distributions along the mitogenome exon phylogenetic tree, South Africa has the broadest distribution, with strains represented in all major clades and sub-clades of the phylogeny (Figure 4). Other countries with broad phylogenetic representations include Botswana and Brazil. The Asian samples are primarily clustered into the VNI sub-clades (Figure 4). Overall, the geographic distributions on the phylogeny are largely consistent with those identified based on nuclear genome SNPs. We would like to note that due to strain representation bias among geographic regions, the geographic distribution identified here may not be truly representative of those in natural populations of this species.

#### Ecological and Mating Type Distributions on Mitogenome Tree

Of the remaining two characters associated with these strains, mating type and ecological niches, their distributions on the mitogenome exon phylogeny are overall similarly consistent with their nuclear genome SNP-based phylogenetic distributions. Specifically, strains of *MAT***α** are broadly distributed on the phylogenetic tree. The relatively few strains with mating types *MAT***a** and *MAT***α/α** are not clustered but are broadly distributed onto the phylogeny, often with identical mitogenome exon sequences to those of *MAT***α** strains, consistent with the lack of mitogenome sequence differentiation between *MAT***a** and *MAT***α** strains in nature. However, the seven diploid strains of *MAT***a**/**α** and one diploid strain of *MAT***a/a** of the VNB/VNIV hybrid origins all had identical mitogenomes to each other. As mentioned above, these 8 strains were likely derived from the same recent hybridization event between strains of VNB and VNIV. Ecologically, the clinical and non-clinical samples within both the VNI and VNB clades are interspersed with each other on the mitogenome exon phylogeny, with strains from different ecological niches often share identical mitogenome exon sequences (Figure 4). The exceptions are the diploid VNB/VNIV hybrids and the VNII isolates where only strains from humans and in the case of VNII, four strains from diseased cats, were included for analyses. Thus, in these two clades, there was no environmental strain for mitogenome comparison.

#### Comparisons of Intron Phylogeny With Their Corresponding Exon Phylogeny

In this sample of 184 strains *C. neoformans*, a total of 12 nucleotide sites located within five mitochondrial genes were found to contain introns. The detailed intron distribution for each strain is shown in Supplementary Table S2 and Figure 4. Some introns were found in only a few isolates while others were found in most of the isolates. For example, the first intron located in *COX1* gene (i.e., the intron closest to the 5′ end of the *COX1* gene), COX1i1, was found in only three strains with one from the VNII2 sub-clade and two representing VNII/VNB hybrids. On the other hand, the ND1i intron was found in all but four strains (three of them in the VNB2 sub-clade and one in the VNII1 sub-clade). Similar to the ND1i intron, intron COBi1 was broadly distributed along the phylogenetic tree, found in representative strains of all major clades (Figure 4). Most of the remaining introns showed some biases in distribution on the mitogenome exon phylogeny (Figure 4). The details of which will be briefly described below.

At each of the 12 intron-containing nucleotide sites, the introns from all the strains were highly similar to each other. In contrast, DNA sequences from introns at different positions were generally highly divergent from each other (data not shown). Among introns at these 12 positions, 11 contained open reading frames coding for either the GIY-YIG endonuclease (in introns ND1i and LsRNAi4) or the LAGLIDADG endonuclease (in introns COBi1, COBi2, COX1i1, COX1i2, COX1i3, COX1i4, COX2i, LsRNAi2, and LsRNAi3). The only intron without an open reading frame was LsRNAi1. Blast analyses of these intronic sequences revealed that their closest matches in GenBank were all from fungi, with different introns in the *C. neoformans* mitogenomes having the highest E scores to introns from different fungal species (details not shown). Below we describe the phylogenetic comparisons between the introns at each of 12 intron positions and their corresponding exons among the 184 strains.

#### Exon–Introns Phylogenetic Relationships Within the *LsRNA* Gene

As shown in Figure 4 and described above, a total of four nucleotide sites within the mitochondrial *LsRNA* gene contained introns in the analyzed population. The phylogenies based on the *LrRNA* exon sequences and sequences at each of the four introns are shown in Supplementary Figures S1–S4. Two of the four introns, LsRNAi1 and LsRNAi3, were found only in the eight VNB/VNIV diploid strains. Furthermore, all eight strains showed identical exon and intron sequences for both introns LsRNAi1 and LsRNAi3 (Supplementary Figures S1, S3). In addition to the eight VNB/VNIV hybrids, intron LsRNAi2 was also found in all the 21 strains of the VNI2 sub-clade, as well as one strain of VNB1 (Tu401-1) and two VNII/VNB hybrids (MW-RSA852 and CCTP51) (Figure 4 and Supplementary Figure S2). DNA sequence variations among strains within the LsRNAi2 intron separated the strains into three unique alleles, with the VNB1 strain and all the 21 VNI strains belonging to one allele while the other two alleles were represented separately by the VNII/VNB and the VNB/VNIV hybrids respectively, consistent with their phylogenetic separation based on *LsRNA* exon sequence (Figure 4 and Supplementary Figure S2). The fourth intron in *LsRNA*, LsRNAi4, was found only in VNII strains and the two VNII/VNB hybrids (MW-RSA852 and CCTP51) (Figure 4 and Supplementary Figure S4). Indeed, all VNII strains has intron LsRNAi4 except one, IFN80, from South Africa. DNA sequence variations among strains within the LsRNAi4 intron separated the strains into two unique alleles, with all the VNII strains sharing one allele and the two VNII/VNB hybrids sharing a different allele (Figure 4 and Supplementary Figure S4).

#### Exon–Intron Phylogenetic Relationship Within the *COB* Gene

As shown in Supplementary Table S2 and Figure 4, there are two introns in the *COB* gene, with both introns broadly distributed along the mitochondrial exon phylogenetic tree. The phylogenies based on the *COB* exon sequences and sequences at each of the two introns are shown in Supplementary Figures S5, S6. Each of the 184 strains analyzed here has at least one intron in the *COB* gene, with several groups of strains containing both introns (Figure 4). For the COBi1 intron, all strains of VNII clade, all strains in VNI2 sub-clade, some strains in VNI1 subclade, most strains in the VNB clade, and all the VNB/VNIV diploid hybrids contained this intron. Aside from the patchy distributions within the VNI and VNB clades (Figure 4), there was no obvious phylogenetic incongruence between the *COB* exon phylogeny and the COBi1 intron phylogeny, with both the exon and intron sequences highly similar to each other with few nucleotide differences separating them (Supplementary Figure S5).

For the COBi2 intron, all strains of VNI contained it while VNII and VNB strains showed patchy distributions. Indeed, only one strain in the VNII clade has this intron. The hybrids also showed patchy distributions, with most of them containing the intron. Similar to the comparison between *COB* exon phylogeny and COBi1 intron phylogeny, we found no obvious phylogenetic incongruence between the *COB* exon phylogeny and COBi2 phylogeny (Supplementary Figure S6).

#### Exon–Intron Phylogenetic Relationships Within the *COX1* Gene

As shown in Figure 4 and Supplementary Table S2, a total of four nucleotide sites within the *C. neoformans* mitochondrial *COX1* gene contained introns in our sample. The phylogenies based on the *COX1* exon sequences and sequences at each of the four introns are shown in Supplementary Figures S7–S10. One intron COX1i1 was only found in three phylogenetically closely related strains and the intron sequences from two of them, MW-RSA852 and CCTP51, were identical to each other, consistent with their *COX1* exon sequence identity (Supplementary Figure S7). The third strain IFN80 had different COX1 exon and COX1i1 intron sequences from the above two strains (Supplementary Figure S7). Compared to intron COX1i1, introns COX1i2 and COX1i3 have broader and very similar distributions to each other (Figure 4 and Supplementary Figures S8, S9). Both the COX1i2 and COX1i3 introns were found in all the analyzed VNII strains but were absent in all the analyzed VNI and VNB strains. In addition, one diploid VNII/VNB hybrid strain PMHc1045.ENR.STOR contained both COX1i2 and COX1i3 introns. Interestingly, two other hybrids of VNII/VNB, MW-RSA852 and CCTP51, contained only the COX1i3 intron but not the COX1i2 intron. No sequence variation was observed among the COX1i2 alleles (Supplementary Figure S8) and for the COX1i3 intron, all VNII strains shared identical DNA sequences (Supplementary Figure S9). However, the two hybrids had slightly different intron COX1i3 sequences from those of VNII strains (Supplementary Figure S9). Different from those of the other three introns within the *COX1* gene, only one strain of VNB (SA8961) and a subset of the VNI strains contained the COX1i4 intron. Furthermore, the COX1i4 introns in all the VNI strains had identical nucleotide sequences (Supplementary Figure S10). Overall, we observed very limited sequence variations among alleles for any of the four *COX1* introns, especially for those in the same clade and/or sub-clade as identified based on their nuclear genome SNPs and mitogenome exon sequences (Supplementary Figures S7–S10).

#### Exon–Intron Phylogenetic Relationship Within the *COX2* Gene

As shown in Figure 4 and Supplementary Table S2, one intron was found within *COX2*. No strain within VNI was found to contain this intron. Within the VNII clade, all strains of the VNII1 sub-clade contained the intron while none of the strains within the VNII2 sub-clade contained the intron (Figure 4). However, the pattern is different for strains in the VNB clade with a portion of strains in both VNB1 and VNB2 sub-clades having the intron while other don’t. Phylogenetic analyses between the *COX2* exon phylogeny and the COX2i intron phylogeny revealed very limited exon sequence variations but a large number of intron sequence types (Supplementary Figure S11). Interestingly, the COX2i intron phylogeny matched well the concatenated mitogenome exon phylogeny, with introns in VNII strains, VNB strains, and the diploid VNB/VNIV hybrids all clustered separately from each other (Figure S11). This result is consistent with the early acquisition of the COX2i intron during *C. neoformans* evolution, followed by specific losses in individual clades and sub-clades.

#### Exon–Intron Phylogenetic Relationship Within the *ND1* Gene

All analyzed *C. neoformans* strains in this study except four contained the ND1i intron. Of the four strains without the intron, one belonged to the VNII1 sub-clade (strain C48), two belonged to the VNB2 sub-clade (V2 and V31), and one was a VNB/VNB diploid (MMRL2445) (Figure 4 and Supplementary Table S2). Comparisons between the *ND1* exon phylogeny and the ND1i intron phylogeny revealed no exon sequence variation among the strains within *ND1* but a large number of intron sequence types (Supplementary Figure S12). However, the ND1i intron phylogeny matched well with the concatenated mitogenome exon phylogeny, with introns in VNII strains, VNB strains, and the diploid VNB/VNIV hybrids all clustered separately from each other. This result is consistent with the early acquisition of the ND1i intron during *C. neoformans* evolution, followed by specific loss(es) in the four strains or their ancestor(s).

### Evidence for Mitogenome Recombination

Our phylogenetic analysis based on the concatenated mitochondrial exon sequences demonstrated that the eight diploid VNB/VNIV hybrids from South Africa contained mitogenomes distinctly different from both the reference VNIV (*C. deneoformans*) strain B-3501A and all the remaining *C. neoformans* samples (including all VNB strains; Figure 4). To investigate the potential origins of the mitogenomes of these eight strains, we compared each of their 17 protein- and rRNA- coding gene sequences individually with each other and with those from the B-3501A and all the other 176 *C. neoformans* strains. Our analyses revealed that all eight VNB/VNIV hybrids had identical concatenated exon sequences to each other. However, depending on the specific gene, their exon sequences may be identical or closely related to either the known VNB strain(s) or the B-3501A strain. Specifically, three (*RPS3, LsRNA*, and *COX3*) of the 17 coding genes in these eight strains had identical or highly similar sequences (>99% sequence identity) to the B-3501A alleles but were distinctly different from all the other 176 *C. neoformans* strains (<93% sequence identity). In contrast, 13 of the 17 coding genes (genes *ATP6, ATP8, ATP9, COB, COX1, COX2, ND1, ND2, ND3, ND4L, ND5, ND6*, and *SsRNA*) in these eight strains had allelic sequences identical or highly similar to the alleles in the 176 *C. neoformans* strains (>99% sequence identity at each locus). Interestingly, in the *ND4* gene, the first 373 nucleotides of the eight hybrids were identical to the B-3501A allele while nucleotides from 465 to 1452 bp (the remaining part of the gene) were identical or highly similar (>99%) to most alleles of the other 176 *C. neoformans* strains. The intermediate nucleotides between those two regions, from 374 to 464 bp of the *ND4* gene, corresponding to 4599–4691 nt of the published H99 mitogenome (GenBank accession number NC004336), were identical among all the strains we analyzed, including B-3501A, H99, the eight VNB/VNIV hybrids, and all the remaining 176 *C. neoformans* isolates. This region contained one recombination breakpoint.

**FIGURE 5:**
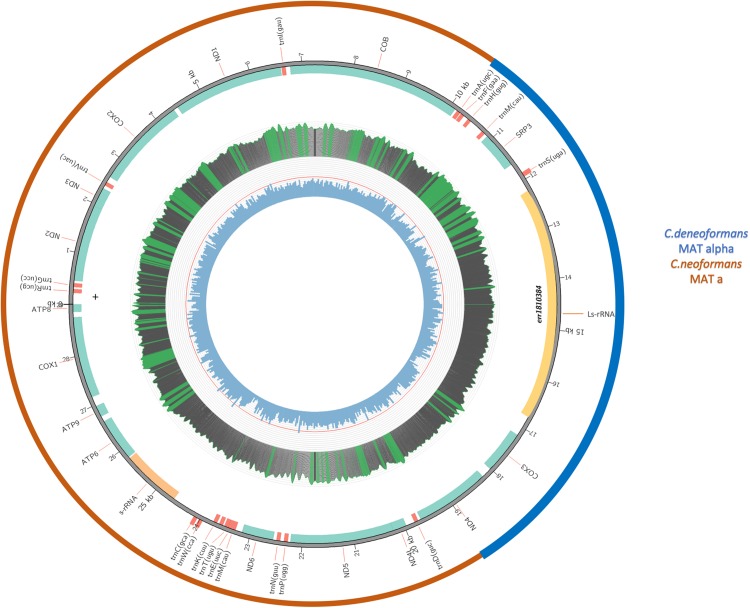
A schematic diagram showing the composition of the eight VNB/VNIV hybrid mitogenomes. The inner circle indicates GC content, with the red line indicating 50% GC content. Middle circle indicates nucleotide coverage in sequence reads. For this sample, the coverage ranges from 5000 to 8000x. Green means the coverage is over top quartile; red means lower than 20x. The second circle from the outside indicates the locations of individual genes. The outermost circle indicates the likely origins of the mitogenome from either *C. neoformans* (Red) or *C. deneoformans* (blue).

Since the three genes (*RPS3, LsRNA*, and *COX3*) showing identical or high sequence similarity to B-3501A were located next to each together in the mitogenome, flanked by *ND4* from one side and *COB* from the other, the intergenic sequence between RPS3 and COB must contain the other recombination breakpoint. Indeed, whole genome sequence comparisons of the assembled mitogenomes of B-3501, H99, and the VNB/VNIV hybrids revealed the other recombination breakpoint to be between 23,058 and 23,059 nt (positions refer to that in the H99 genome), at the end of *COB* gene. [Due to the large number of files and large file sizes in the phylogenetic analyses of the 17 coding genes, the detailed graphs and data of the analyses are not presented but they are available from the authors.] Figure 5 is a schematic diagram showing the composition of the eight VNB/VNIV hybrid mitogenomes.

## Discussion

In this study, we analyzed the mitogenome variations among 184 strains of *C. neoformans*, the most prevalent human pathogen of the *Cryptococcus* species complex. Our analyses identified large genome size and intron number variations among the analyzed strains. In addition, the concatenated mitogenome exon sequences revealed strain relationships largely consistent with those derived from nuclear genome SNPs. However, inconsistencies were also observed, especially among hybrids from between different lineages. Overall, there was little evidence for mitogenome-based strain relationships according to geographic origin or ecological niches. Interestingly, unambiguous evidence for mitogenome recombination was found for several strains. Below we discuss the relevance and potential implications of our results.

### Mitogenome Size and Intron Number Variation

Previous studies have shown that fungal mitogenomes can vary in size from ∼12 to over 200 kb, a difference of over 20 folds (Sandor et al., 2018^3^; Zhang et al., 2020). The mitogenome sizes for strains of *C. neoformans* analyzed here are within the range of published fungal mitogenome sizes, but toward the small end. However, most of previous studies had relatively few, or in some cases only one, strain from each species. To our knowledge, the 184 mitogenomes analyzed here represented the largest number of fungal mitogenomes from within the same species in a single study. Our results demonstrated that within *C. neoformans*, the mitogenome sizes among strains can vary by over 20%.

Despite the relatively large mitogenome size variation among strains, all the mitogenomes of the 184 strains contained the same number of protein-coding genes, ribosomal RNA genes, and tRNA genes; and in the same order. The mitochondrial genes found in these *C. neoformans* strains were also commonly found in most of the published fungal mitochondrial genomes. The main contributor to the mitogenome size differences among *C. neoformans* strains was introns (both intron number and summed intron lengths), also similar to previous comparisons among fungal species (Sandor et al., 2018; Zhang et al., 2020). These comparisons suggest that the main mechanisms driving mitogenome size variation among strains within fungal species are likely similar to those driving mitogenome size variation among fungal species. In both cases, intron gains and losses are primarily responsible for the variations in fungal mitogenome sizes. The open reading frames in 11 of the 12 introns encoding endonucleases are consistent with the mobility of these introns (Yan et al., 2018). However, our study also found that intergenic regions and exon sequences contributed significantly to mitogenome size variations in *C. neoformans*.

In this study, *C. neoformans* strains from most geographic regions and ecological niches showed a range of mitogenome sizes and a diversity of intron distributions. The lack of a clear geographic and ecological pattern for the *C. neoformans* mitogenomes is consistent with the organism’s ubiquitous distributions in a variety of ecological niches and with its long-distance dispersal ability. As suggested in other studies (e.g., Xu et al., 2000b; Rhodes et al., 2017; Samarasinghe et al., 2019), both natural factors such as wind and bird migrations and anthropogenic factors such as human travel and commercial trade could have contributed to the gene flow of *C. neoformans* and the observed lack of geographic pattern for mitogenome size and intron distributions.

However, due to the biased/limited samples from certain geographic regions and ecological niches, we would like to note that more extensive sampling might reveal certain region-specific differences in mitogenome size and intron number distributions. For example, the Far East Asian population of *C. neoformans* is dominated by ST5 and its clonal derivatives based on MLST at seven nuclear loci (Chen et al., 2018). The two strains analyzed here, CCH193 from China and Jp1088 from Japan, have identical mitogenome sequences and very similar nuclear genome sequences. It’s likely that most, if not all, ST5 strains from Far East Asia have the same mitogenome size and intron number. Genome sequences from additional strains are needed in order to confirm this hypothesis.

### Mitogenome Exon Phylogeny

Aside from genome size and intron number variations among strains, single nucleotide polymorphisms were also found among the 184 mitogenomes. The strain relationships and their clade affiliations as revealed by mitogenome SNPs were largely consistent with those inferred based on nuclear genome SNPs (Figure 4). Overall, the results indicate that through most of the evolutionary history of this species, these clades and sub-clades have been evolving independently. However, phylogenetic analyses of mitogenome exon SNPs revealed several novel features that are different from those based on nuclear genome SNPs. For example, *C. neoformans* strains were clustered into additional mitogenome sub-clades (VNI1, VNI2, VNII1, VNII2, VNB1, and VNB2), some of which were different in their clustering patterns from those inferred based on their nuclear genome SNPs (Figure 4).

One strain showing different clustering pattern between the mitochondrial and nuclear genomes is strain RCT21 from South Africa. This strain showed a nuclear genome of the VNB type, almost identical to the nuclear genome sequence of another strain of VNB, RCT26 (Rhodes et al., 2017). However, RCT21 has a mitogenome of the VNI1 type, with identical mitogenome exon sequences to a large number of VNI1 strains, but very different from that of RCT26 which were clustered into VNB1, as expected (Figure 4). At present, the detailed process for generating RCT21 is not known. One possibility for the inconsistent grouping between the nuclear and mitochondrial genomes of RCT21 was that it might have been derived from a mating event between a VNI and a VNB strain, with the mating product, a dikaryotic hypha, containing two haploid parental nuclei but inheriting the mitogenome of the VNI parent. However, during hyphal extension of the dikaryon and before sexual reproduction, asexual spores such as conidia or chlamydospores containing one of the two haploid nuclei may form. If the nucleus in the haploid asexual spore was that of the original VNB parent, a recombinant like RCT21 could be formed. Indeed, this process was used to generate a laboratory strain containing the nuclear genome from one parent but mitochondrial genome from another in an early study (Yan and Xu, 2003). However, as revealed by the combined results of the Rhodes et al. (2017) study and this study, most inter-clade hybrids contained nuclear genes from both parental clades but mitochondrial genome from one parent.

### Mitogenome Recombination

Our sequence analyses revealed that the eight VNB/VNIV hybrids from South Africa contained mitogenomes with signature sequences from both the VNB and the VNIV clades. In addition, we were able to identify the regions where recombination breakpoints were most likely located (Figure 5). Laboratory investigations of *C. neoformans* (serotype A) and *C. deneoformans* (serotype D) crosses revealed that their mitogenomes were uniparentally inherited from the *MAT***a** parent (Xu et al., 2000a). In addition, analyses of natural serotype AD hybrids were found to contain mitochondrial DNA also from the *MAT***a** parent (Yan and Xu, 2003). Specifically, a serotype AD strain having its *MAT***a** locus from a serotype A parent and its *MAT***a** locus from a serotype D parent, i.e., an **a**AD**a** hybrid, would have a mitochondrial genotype of the serotype A parent. Conversely, a serotype AD strain having its *MAT***a** locus from a serotype D parent and its *MAT***a** locus from a serotype A parent, i.e., an **a**AD**a** hybrid, would have a mitochondrial genotype of the serotype D parent. The recombinant mitogenomes here of the eight VNB/VNIV hybrids seemed different from those earlier studies where uniparental mitogenome inheritance was shown.

At present, the mechanisms for the difference between these eight hybrids and earlier investigated hybrids are not known. However, there are a couple of possibilities. In the first, the mating condition(s) under which the eight VNB/VNIV hybrids were generated might have been different from the laboratory conditions used to generate laboratory serotype AD hybrids and different from those of the other natural serotype AD hybrids analyzed earlier. Previous studies have shown that under stressful conditions, such as UV irradiation and high temperature, during *C. neoformans* mating, biparental mitochondrial inheritance could be common and that progeny could have recombinant mitochondrial genotypes (Yan et al., 2007). The second possibility might be due to the differences in the markers analyzed between this study and earlier studies. Earlier studies of mitochondrial inheritance in *C. neoformans* were primarily based on 1–2 PCR-RFLP markers, targeting one or two single nucleotide polymorphisms (Xu et al., 2000a; Yan and Xu, 2003). Analyzing additional markers in those crosses might have revealed mitogenome recombination events.

### Mitogenome Intron Distributions

We observed variations in the number of strains containing each of the 12 introns among the 184 strains. None of the 12 introns were distributed in all the 184 strains and none of the 184 strains were found to have all 12 introns. However, some of the introns (e.g., ND1i and COBi2) were more frequently found than others (e.g., COX1i1) among the 184 strains. Despite the overall broad distributions for most of the introns, some introns showed biased distributions at the clade or sub-clade levels as defined based on the concatenated mitochondrial exon sequences. If we use strain B-3501A of the closely related species *C. deneoformans* B-3501A as the mitogenome phylogeny outgroup for *C. neoformans*, the ancestral intron state of the *C. neoformans* would be containing nine of the introns (LsRNAi1, LsRNAi2, LsRNAi3, COBi1, COBi2, COX1i2, COX1i3, COX1i4, and ND1i). Based on this assumption, as none of the 176 strains of *C. neoformans* contained introns LsRNAi1 and LsRNAi3, these two must have been lost in the ancestor of *C. neoformans*. Similarly, intron LsRNAi2 was lost in the ancestor of *C. neoformans* but was regained by the ancestor of the VNI2 sub-clade. As for intron LrRNAi4, it was likely newly gained by the VNII clade. In contrast to the single gain and lost events for the four *LsRNA* introns, the two introns in *COB*, COBi1 and COBi2, were likely gained and/or lost multiple times. The COX1i1 intron was similar to that of LsRNAi4, likely gained recently once by an ancestor of VNII but was lost again in some of the descendants. Introns COX1i2, COX1i3, COX1i4, and COX2i were likely lost once and/or re-gained once or twice during the evolution of *C. neoformans*. Finally, the simplest explanation for the ND1i distribution is that it lost twice, one in the VNB2 sub-clade and the other for strain C45 of the VNII1 sub-clade. Based on the high sequence similarity among alleles within each of the low-frequency introns, most of the intron gains (or regains) seemed recent (Supplementary Figures S1–S12).

We note that the above inferred intron gains and losses were based on a single strain of a closely related species *C. deneoformans* as the outgroup. If other strains with different intron distributions were used as the outgroup and for ancestral state inference, the number of gain/loss events would differ. At present, the ecological, evolutionary and molecular mechanisms driving the mitogenome intron distributions are largely unknown. A recent study revealed that mutation in the sex-determining gene *SXI1*a could lead to the rapid spread of mitochondrial introns in *C. deneoformans* (Yan et al., 2018). However, regardless of the potential mechanisms, the highly skewed distributions of intron(s) toward certain clade and sub-clade suggest a potentially fast and efficient method for their identification, based on 1–3 PCR reactions. For example, the presence of both intron LsRNAi4 and COX1i2 would be indicative of strains in the VNII clade, and a further assay based on intron COX2i would allow the separation of VNII1 (the presence of this intron) from VNII2 (the absence of this intron). Similarly, the presence of both LsRNAi2 and COX1i4 would be indicative of their VNI2 subclade affiliation. The usefulness of the suggested tests needs experimental validation, including more samples, especially those from under-represented clades, geographic regions, and ecological niches, for analyses.

## Conclusion

In this study, we assembled and analyzed the mitogenomes of a large number of strains of the human fungal pathogen *C. neoformans*. Our analyses identified variations among strains in both genome size and intron number. In addition, the mitogenome phylogeny based on concatenated exon sequences revealed the potential evolutionary relationships among the 184 strains with regard to their other features such as their nuclear genome affiliations, and their geographic and ecological origins. Our analyses identified several novel insights into the evolution of this organism. Furthermore, our results opened up new avenues of future research. For example, where did the introns in *C. neoformans* originate? How did they invade the mitogenomes of *C. neoformans*? How did *C. neoformans* maintain their mitogenome structure and gene order, despite the seemingly frequent intron gain and loss events? What are the effects of these introns on the adaptation and pathogenicity of *C. neoformans* clades and sub-clades? Will continued anthropogenic activities increase the spread of mitochondrial introns? Answers to these and other related questions could provide additional insights into the evolution and diversification of *C. neoformans* as well as their potential effects on its pathogenicity.

## Data Availability Statement

All datasets generated for this study are included in the article/Supplementary Material.

## Author Contributions

YW retrieved and analyzed the data. JX conceived and guided the study. Both authors contributed to writing the manuscript.

## Conflict of Interest

The authors declare that the research was conducted in the absence of any commercial or financial relationships that could be construed as a potential conflict of interest.

## Footnotes

1 https://rdrr.io/cran/plotly/

2 http://hmmer.org/

3 https://www.ncbi.nlm.nih.gov/genomes/GenomesGroup.cgi?opt=organelle&taxid=4751&sort=Genome

## Supplementary Material

FIGURE S1 Co-phylogenetic tree of *LsRNA* concatenated exons and LsRNAi1.

FIGURE S2 Co-phylogenetic tree of *LsRNA* concatenated exons and LsRNAi2.

FIGURE S3 Co-phylogenetic tree of *LsRNA* concatenated exons and LsRNAi3.

FIGURE S4 Co-phylogenetic tree of *LsRNA* concatenated exons and LsRNAi4.

FIGURE S5 Co-phylogenetic tree of *COB* concatenated exons and COBi1.

FIGURE S6 Co-phylogenetic tree of *COB* concatenated exons and COBi2.

FIGURE S7 Co-phylogenetic tree of *COX1* concatenated exon and COX1i1.

FIGURE S8 Co-phylogenetic tree of *COX1* concatenated exon and COX1i2.

FIGURE S9 Co-phylogenetic tree of *COX1* concatenated exon and COX1i3.

FIGURE S10 Co-phylogenetic tree of *COX1* concatenated exon and COX1i4.

FIGURE S11 Co-phylogenetic tree of *COX2* concatenated exon and COX2i.

FIGURE S12 Co-phylogenetic tree of *ND1* concatenated exon and ND1i.

TABLE S1 Mitogenome size and intron number distributions among nuclear genome-based clades, geographic regions, ecological niches, and mating types.

TABLE S2 Details of all strains analyzed in this study. Included in this table for each strain are strain name, geographic origin, ecological niche, mating type, GenBank accession numbers, mitogenome size, intron number and distributions, summed intron length, summed exon length, and summed intergenic spacer length.
