# Supplementary figures and images for: Mitochondrial Genome Polymorphisms in the Human Pathogenic Fungus Cryptococcus neoformans
Source: Front Microbiol. 2020 Apr 21;11:706. doi: 10.3389/fmicb.2020.00706 (PMC7186387; doi:10.3389/fmicb.2020.00706)

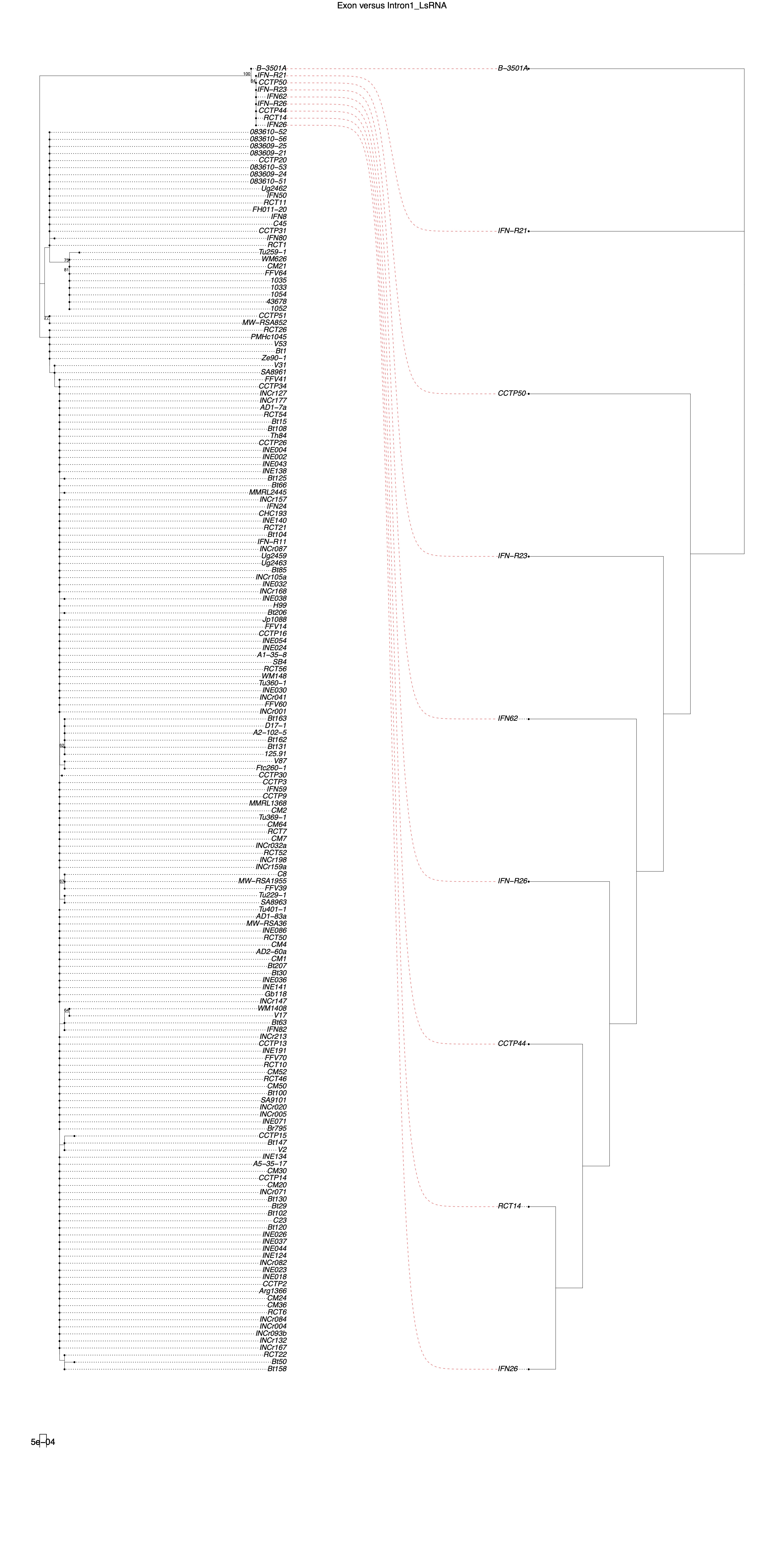

Supplement: FIGURE S1 — Co-phylogenetic tree of LsRNA concatenated exons and LsRNAi1. [file Image_1.JPEG]

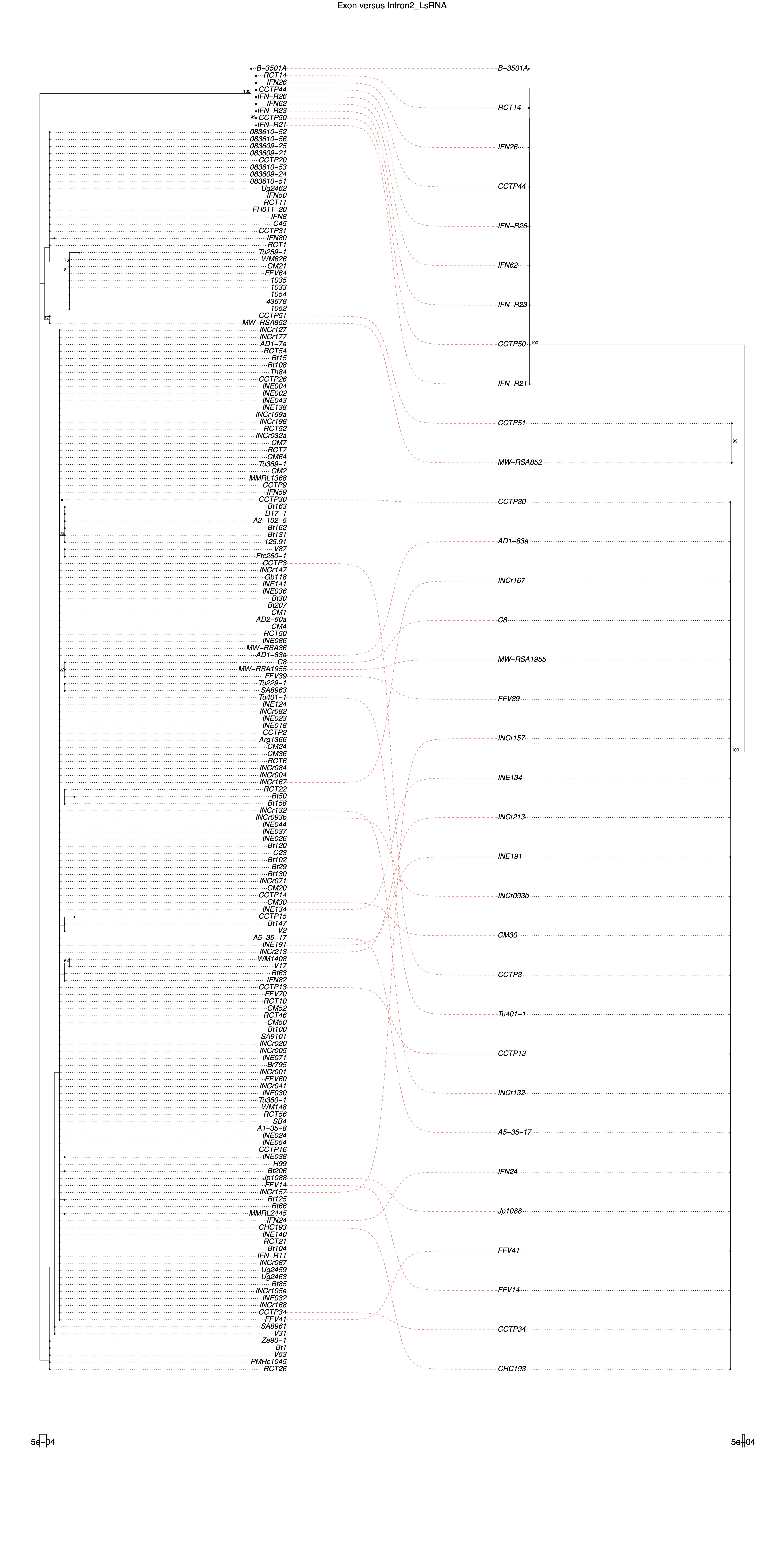

Supplement: FIGURE S2 — Co-phylogenetic tree of LsRNA concatenated exons and LsRNAi2. [file Image_2.JPEG]

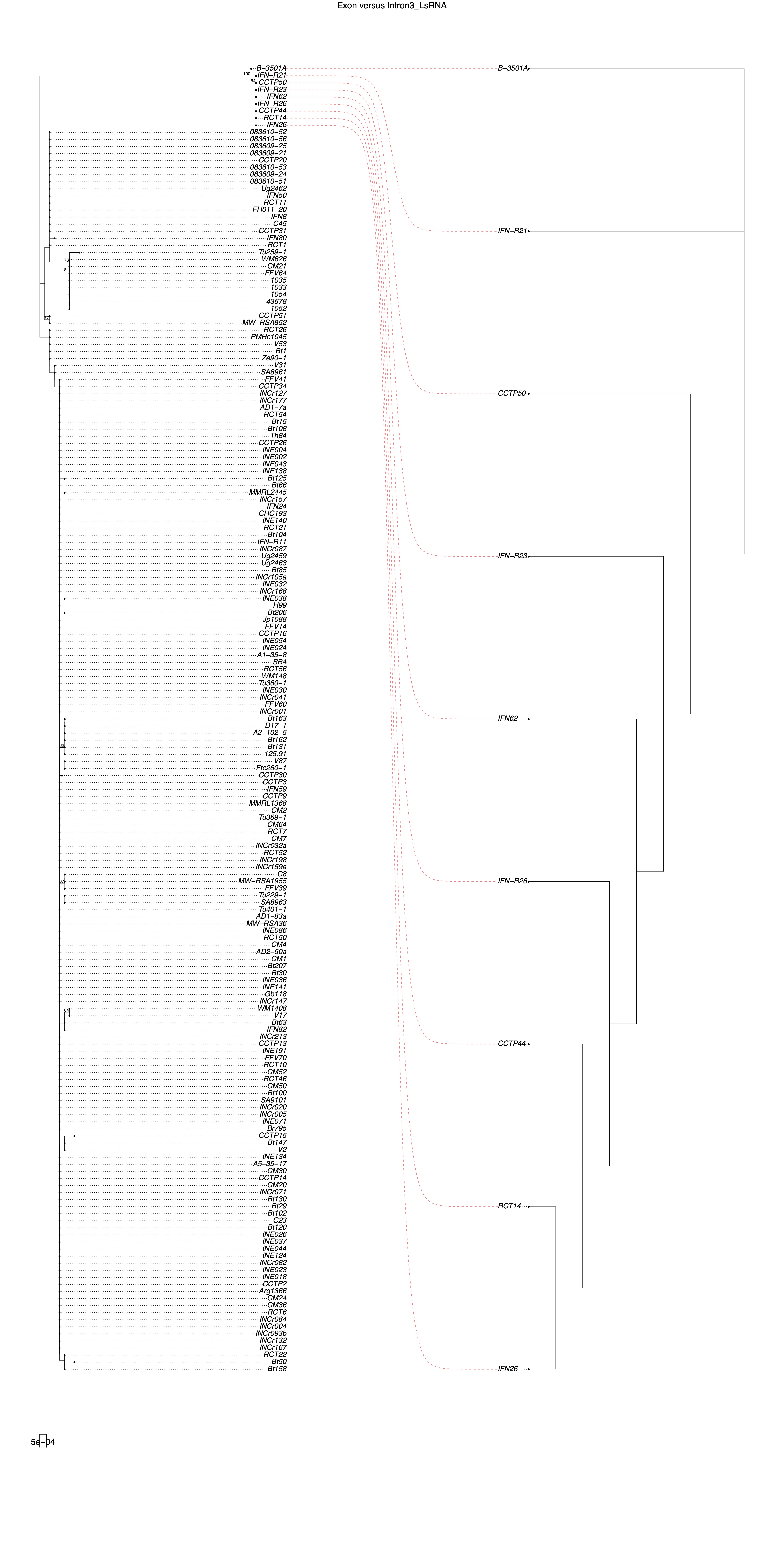

Supplement: FIGURE S3 — Co-phylogenetic tree of LsRNA concatenated exons and LsRNAi3. [file Image_3.JPEG]

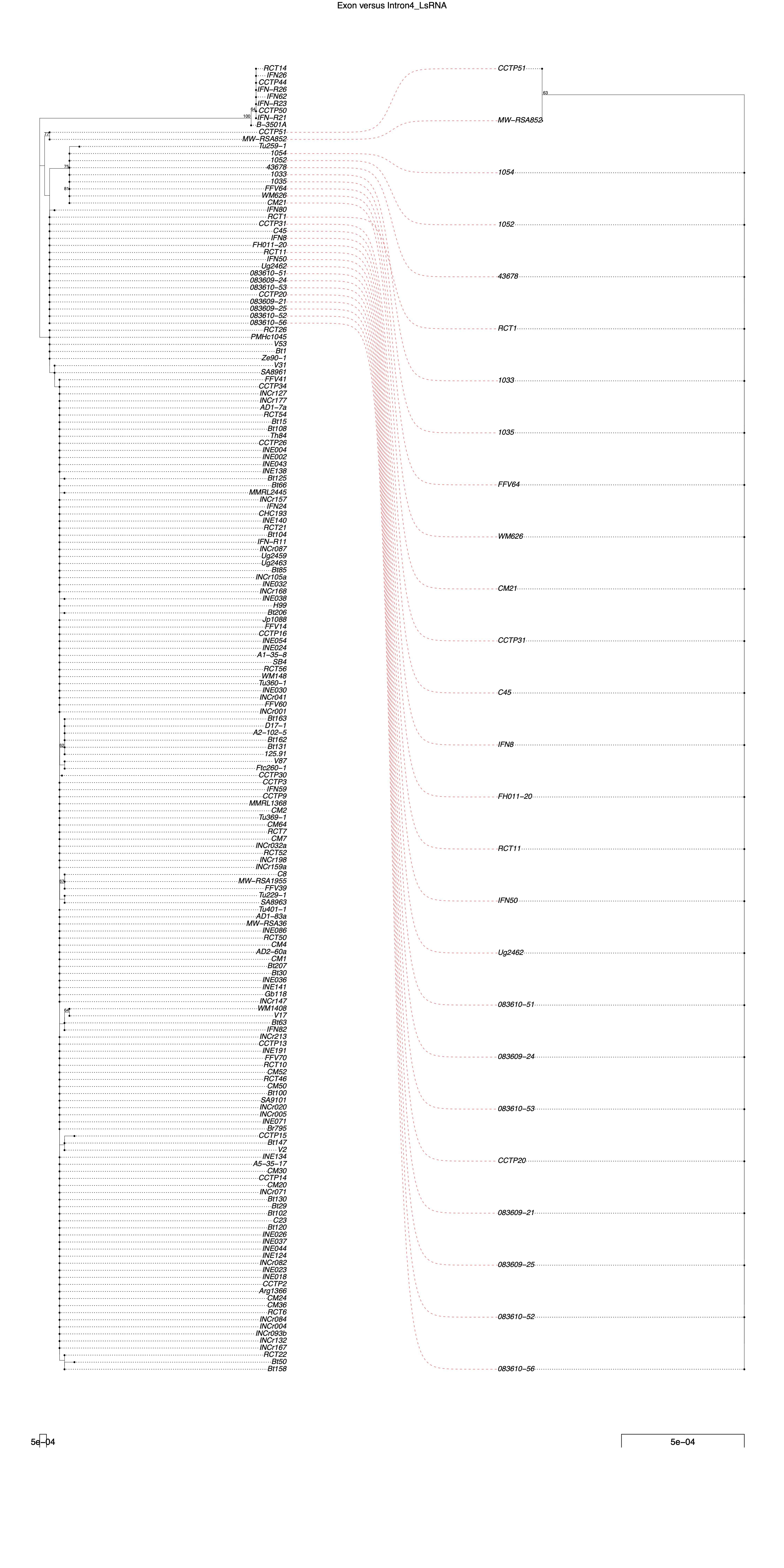

Supplement: FIGURE S4 — Co-phylogenetic tree of LsRNA concatenated exons and LsRNAi4. [file Image_4.JPEG]

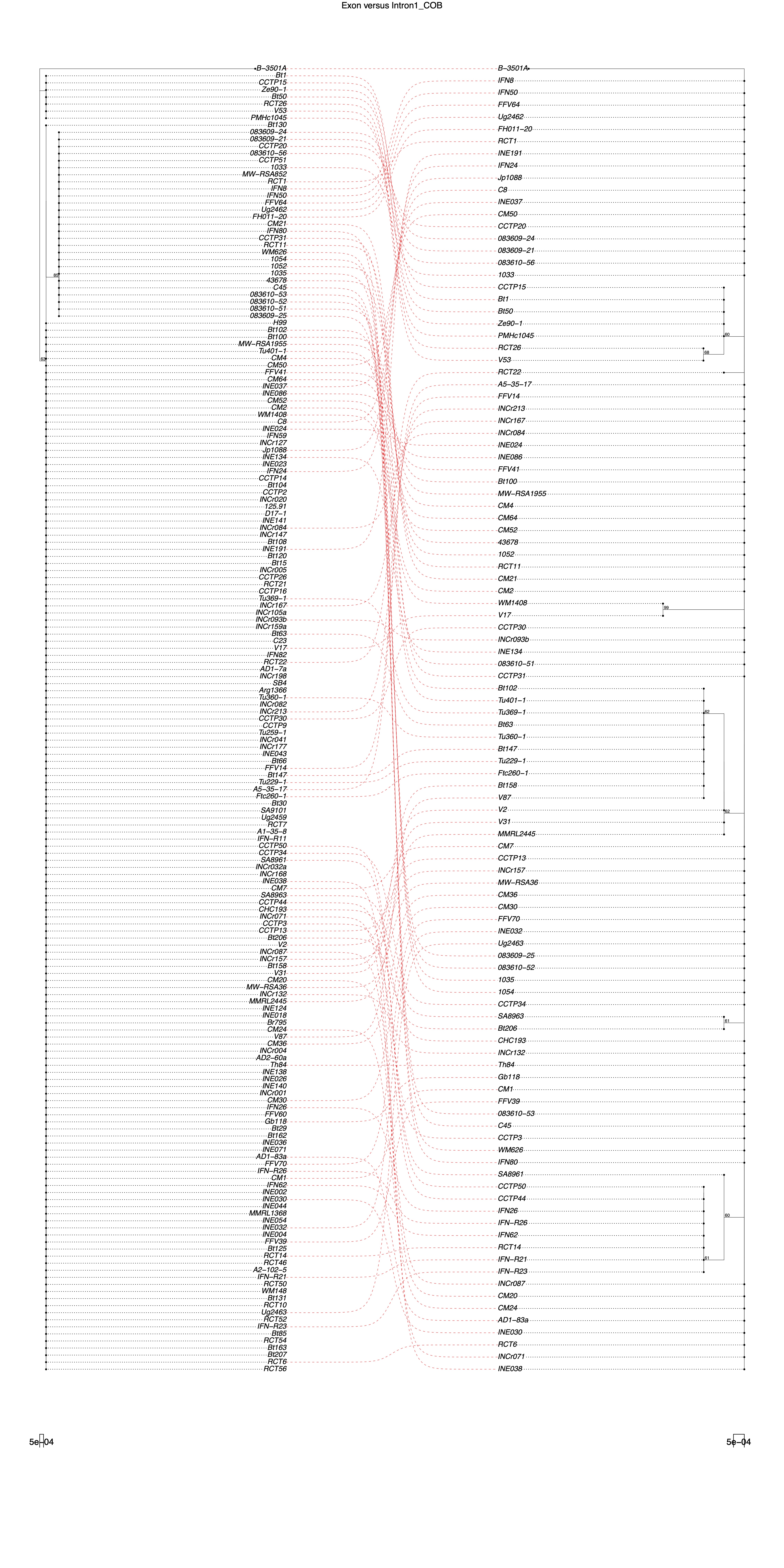

Supplement: FIGURE S5 — Co-phylogenetic tree of COB concatenated exons and COBi1. [file Image_5.JPEG]

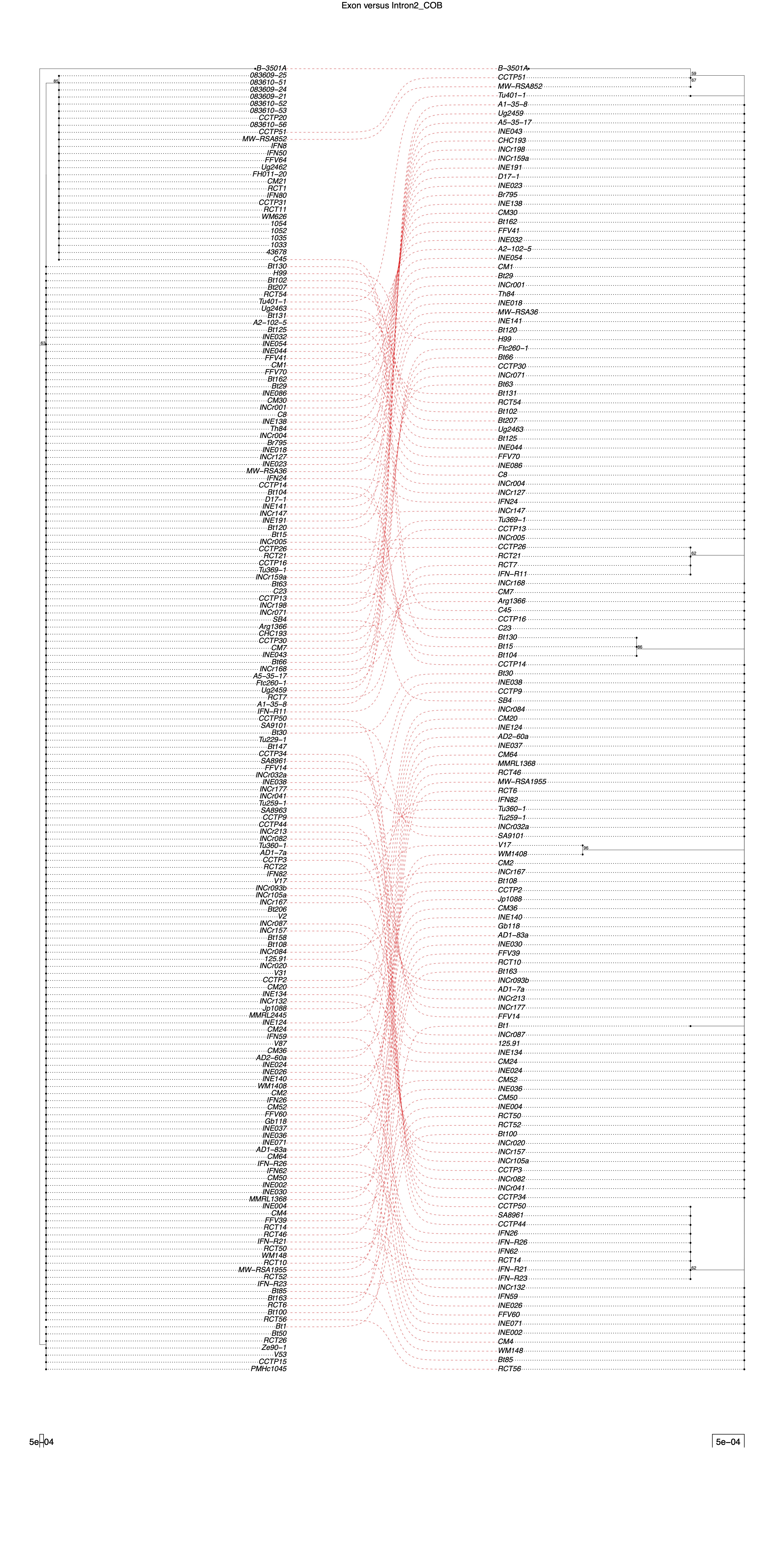

Supplement: FIGURE S6 — Co-phylogenetic tree of COB concatenated exons and COBi2. [file Image_6.JPEG]

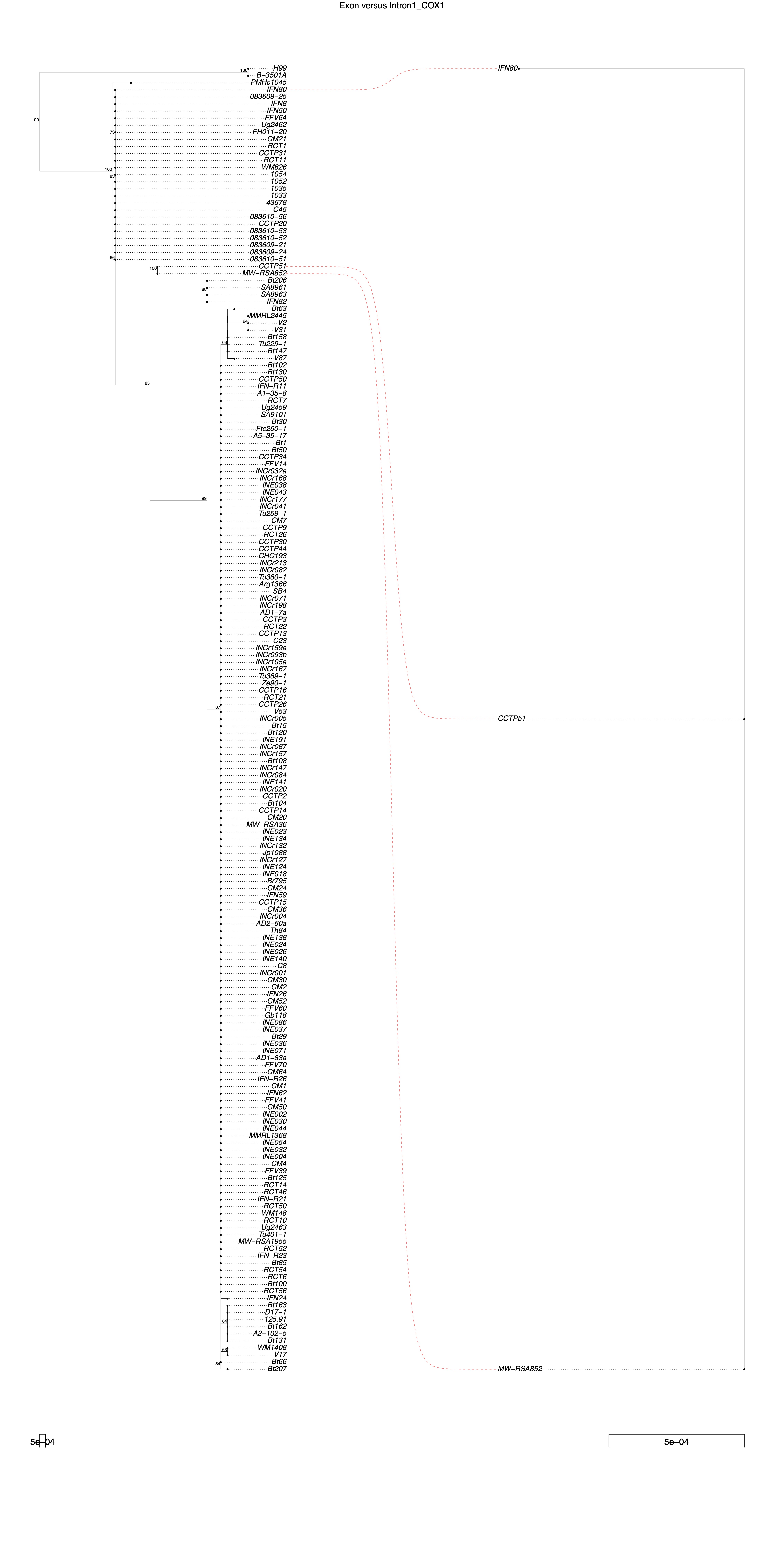

Supplement: FIGURE S7 — Co-phylogenetic tree of COX1 concatenated exon and COX1i1. [file Image_7.JPEG]

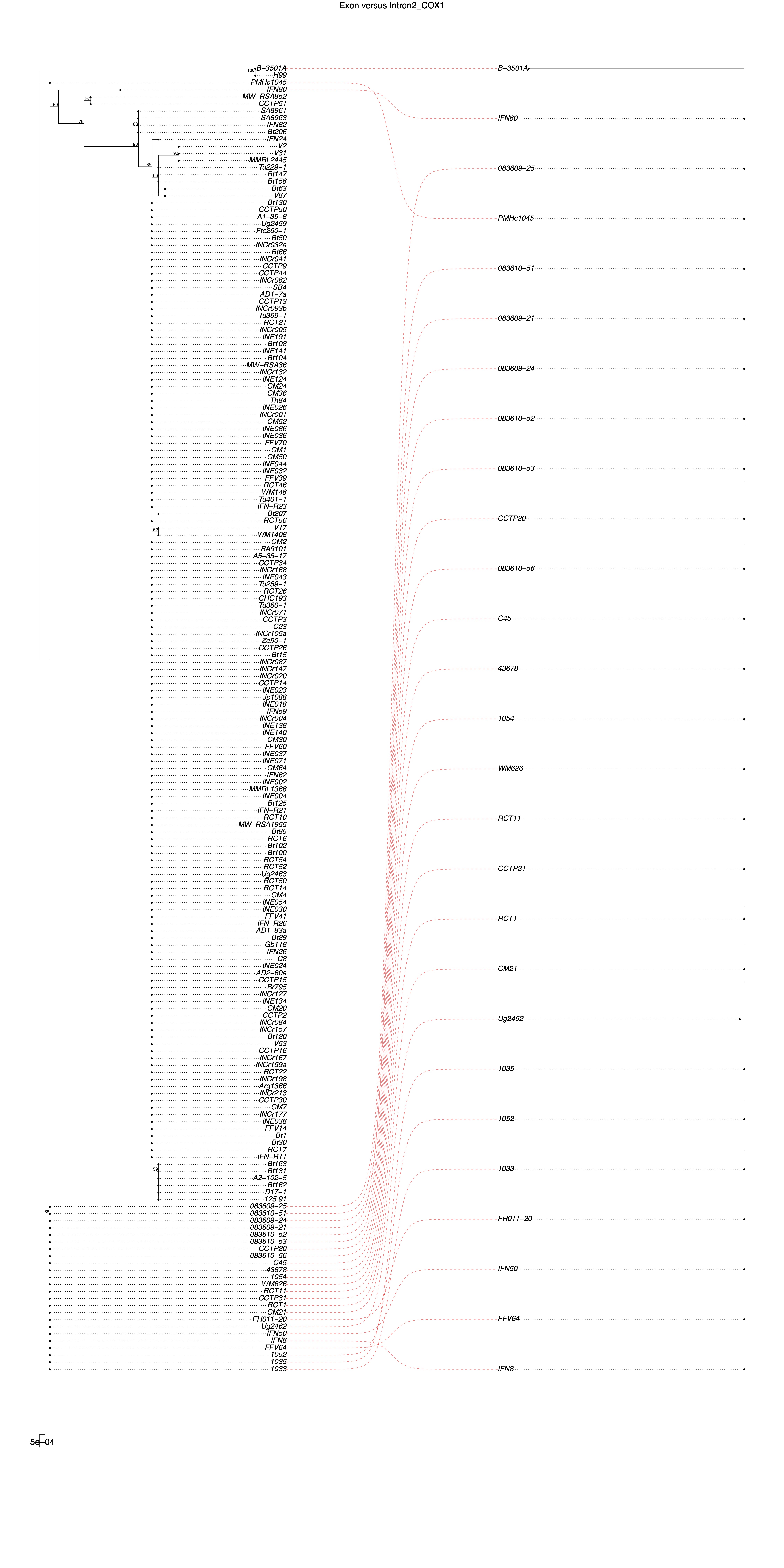

Supplement: FIGURE S8 — Co-phylogenetic tree of COX1 concatenated exon and COX1i2. [file Image_8.JPEG]

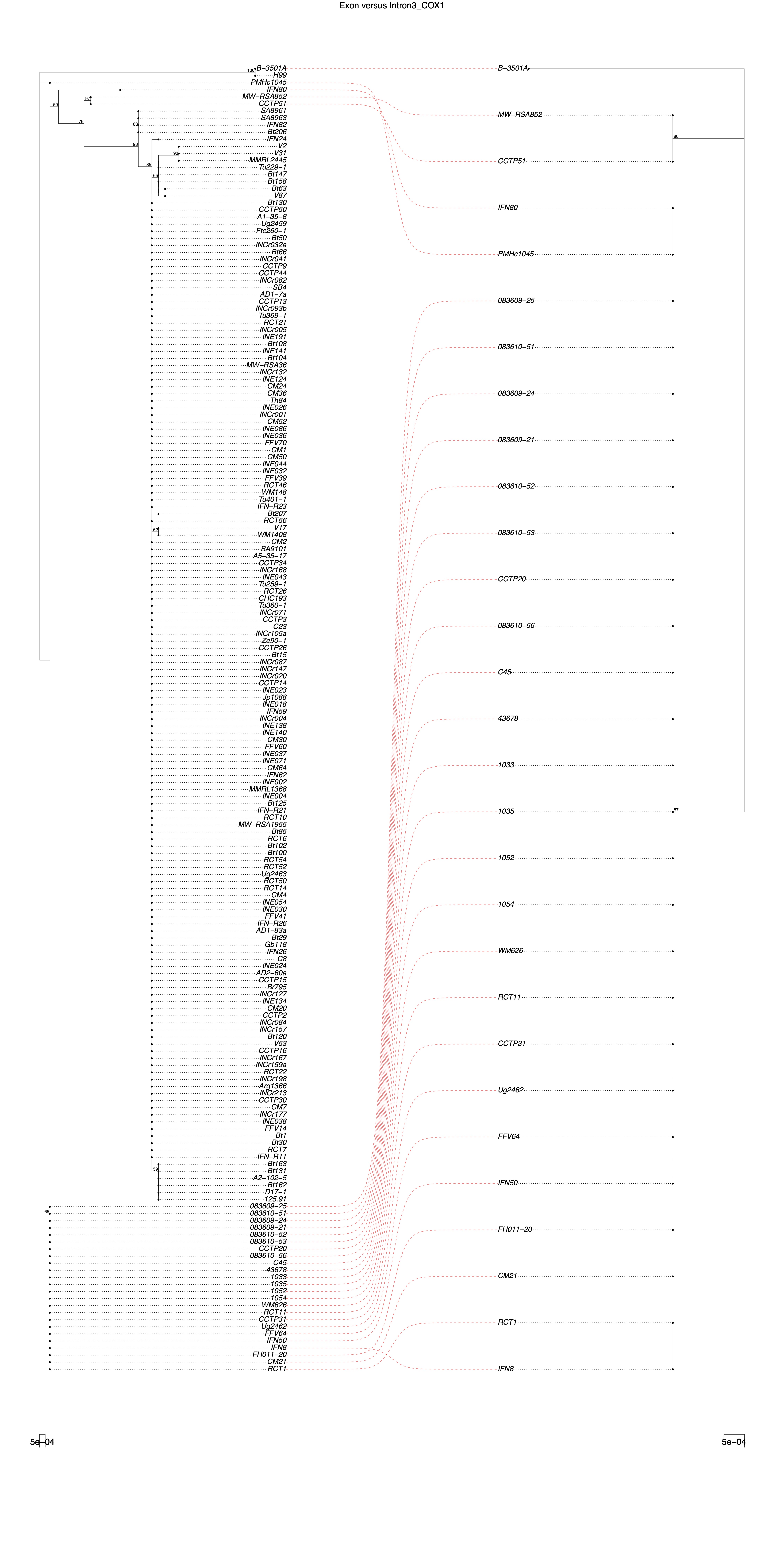

Supplement: FIGURE S9 — Co-phylogenetic tree of COX1 concatenated exon and COX1i3. [file Image_9.JPEG]

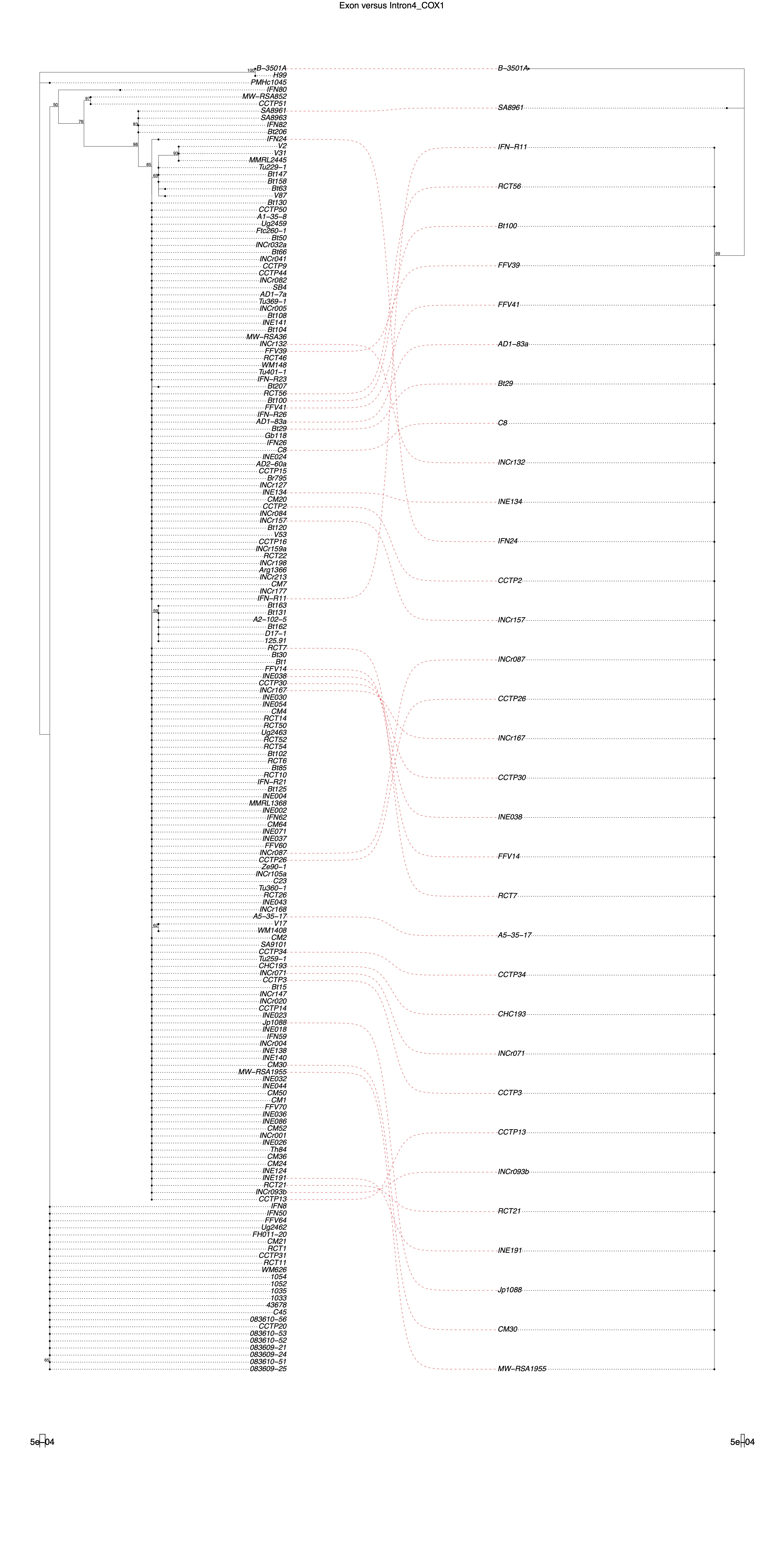

Supplement: FIGURE S10 — Co-phylogenetic tree of COX1 concatenated exon and COX1i4. [file Image_10.JPEG]

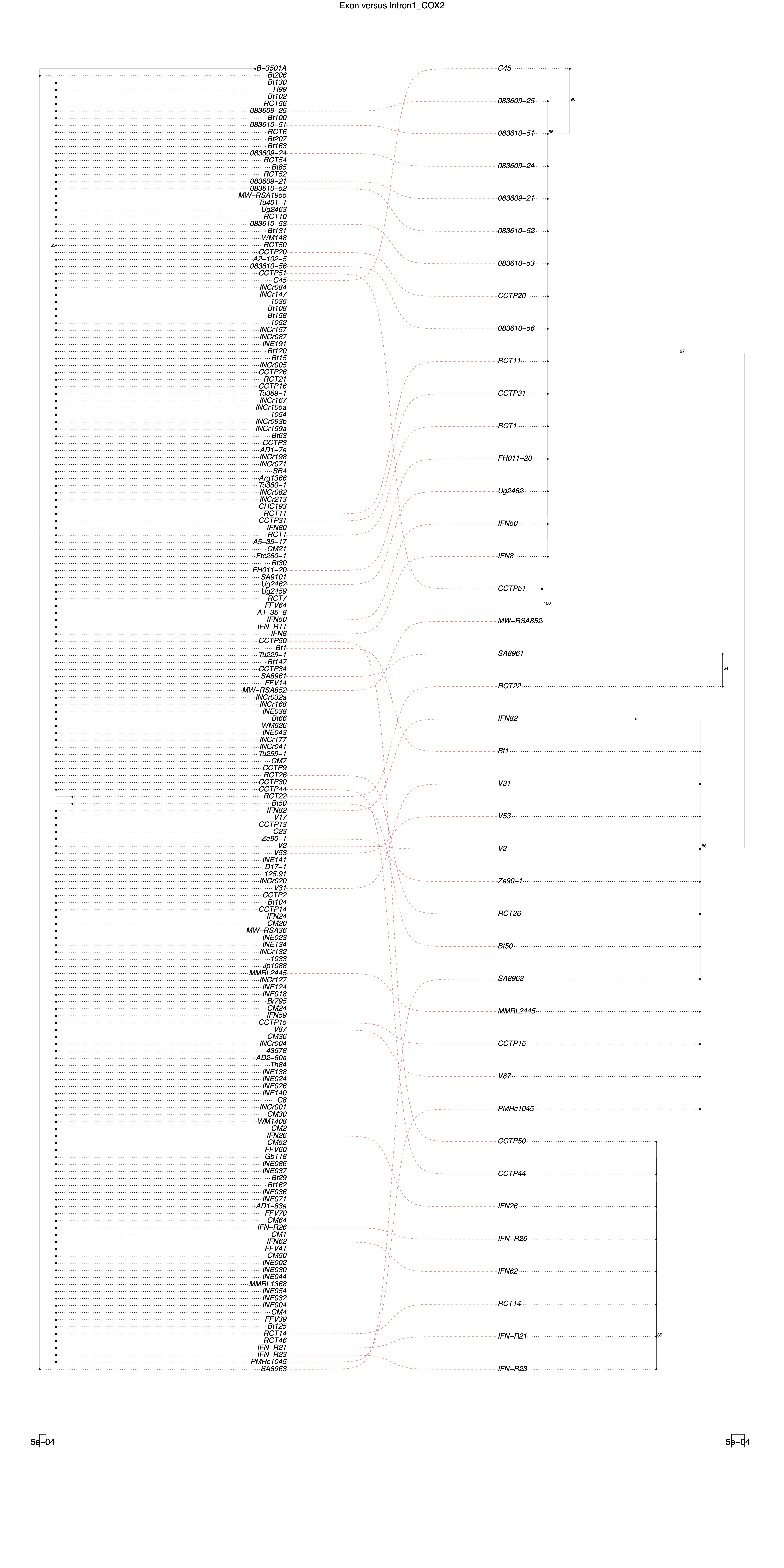

Supplement: FIGURE S11 — Co-phylogenetic tree of COX2 concatenated exon and COX2i. [file Image_11.JPEG]

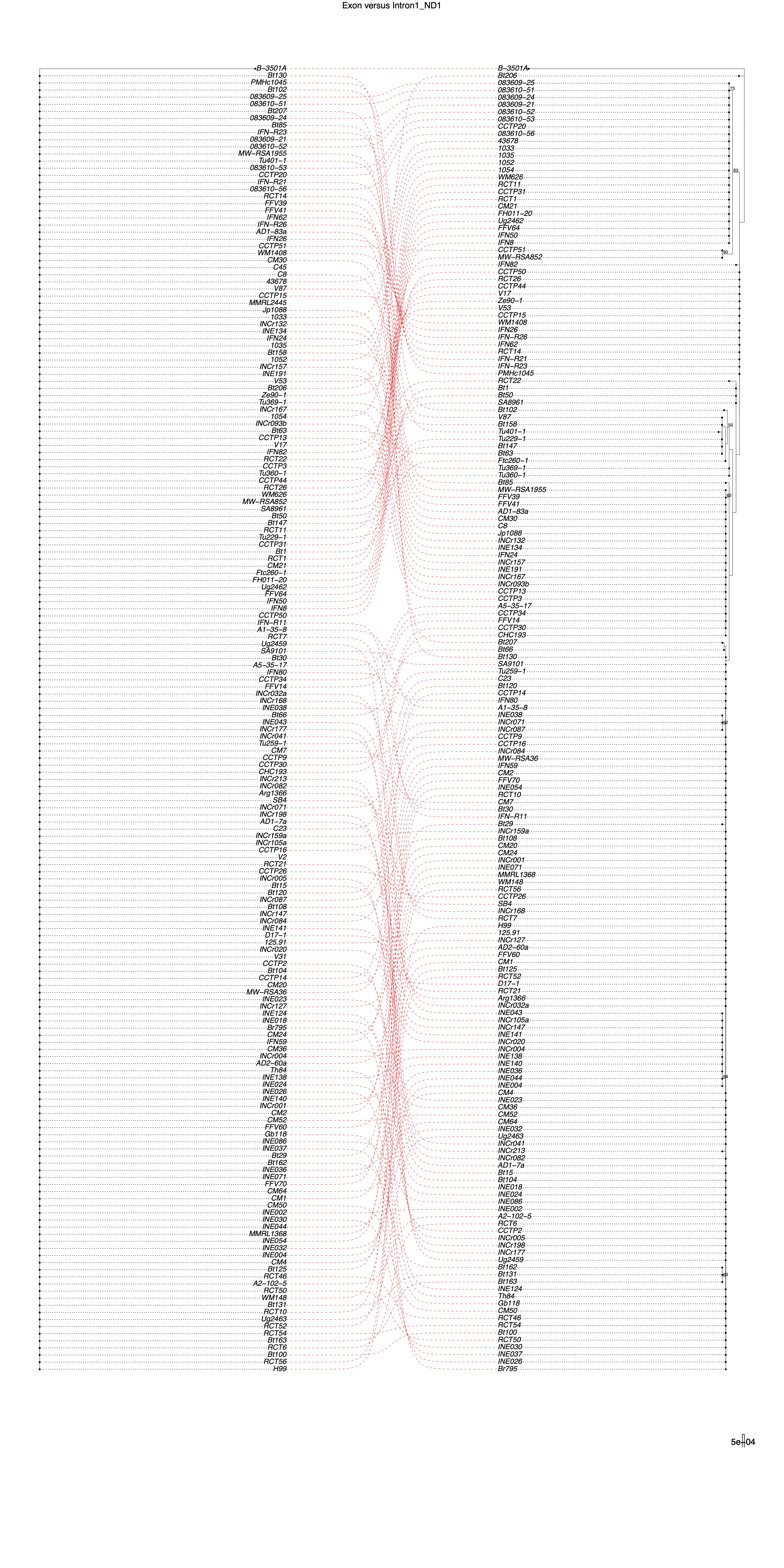

Supplement: FIGURE S12 — Co-phylogenetic tree of ND1 concatenated exon and ND1i. [file Image_12.JPEG]
