# Supplementary material for: Mitochondrial Genome Polymorphisms in the Human Pathogenic Fungus Cryptococcus neoformans
Source: Front Microbiol. 2020 Apr 21;11:706. doi: 10.3389/fmicb.2020.00706 (PMC7186387; doi:10.3389/fmicb.2020.00706)
Supplement: TABLE S1 — Mitogenome size and intron number distributions among nuclear genome-based clades, geographic regions, ecological niches, and mating types. [file Table_1.DOCX]

Table S1. Variations in mitogenome sizes and intron numbers among strains of *Cryptococcus neoformans* clades, countries, mating types and ecological niches.

| **Grouping category** | **Sub-Categories** | **Mitogenome Size Mean +SD**  **(range, if variable) (all in bp)** | **Intron Number Mean + SD**  **(Range)** |
| --- | --- | --- | --- |
| Lineage (Sample size) | Diploid | 25447±500 | 2.3±0.5 |
|  | VNB/VNB (3) | (24740-25818) | (2-3) |
|  |  |  |  |
|  | Diploid | 29002±0 | 7±0 |
|  | VNB/VNIV (8) |  |  |
|  |  |  |  |
|  | Diploid  VNI/VNB (1) | 24901±0 | 2±0 |
|  |  |  |  |
|  | Diploid | 25220.7±495 | 2.3±0.5 |
|  | VNI/VNI (3) | (24868-25920) | (2-3) |
|  |  |  |  |
|  | Diploid | 28923±0 | 5±0 |
|  | VNII/VNB (1) |  |  |
|  |  |  |  |
|  | Diploid | 28932±0 | 5±0 |
|  | VNII/VNII (4) |  |  |
|  |  |  |  |
|  | Haploid | 25069.8±408 | 2.2±0.4 |
|  | hybrid VNI/VNB (5) | (24865-25885) | (2-3) |
|  |  |  |  |
|  | Haploid | 31327±0 | 7±0 |
|  | hybrid VNII/VNB (2) |  |  |
|  |  |  |  |
|  | Haploid | 26083.5±953 | 2.9±0.7 |
|  | VNB (24) | (24740-29154) | (2-5) |
|  |  |  |  |
|  | Haploid | 25808.9±1202 | 2.9±1.1 |
|  | VNI (112) | (24865-28076) | (2-5) |
|  |  |  |  |
|  | Haploid | 29717.6±425 | 5.7±0.5 |
|  | VNII (21) | (28925-30007) | (4-6) |
|  |  |  |  |
| Country (Sample size) | Argentina (2) | 24874±0 | 2±0 |
|  |  |  |  |
|  | Australia (6) | 28253.5±1514 | 4.5±1.1 |
|  |  | (24868-28932) | (2-5) |
|  |  |  |  |
|  | Botswana (30) | 25463.1±945 | 2.6±0.8 |
|  |  | (24740-28923) | (2-5) |
|  |  |  |  |
|  | Brazil (10) | 26395.2±1407 | 2.8±1.2 |
|  |  | (24865-29951) | (2-6) |
|  |  |  |  |
|  | China (1) | 27974±0 | 5±0 |
|  |  |  |  |
|  | Cuba (7) | 29951±0 | 6±0 |
|  |  |  |  |
|  | France (3) | 25903.3±1464 | 3±1.4 |
|  |  | (24868-27974) | (2-5) |
|  |  |  |  |
|  | India (44) | 25644.7±1176 | 2.7±1.1 |
|  |  | (24865-28076) | (2-5) |
|  |  |  |  |
|  | Japan (1) | 27974±0 | 5±0 |
|  |  |  |  |
|  | S. Africa (55) | 27289.3±1957 | 4.3±1.8 |
|  |  | (24865-31327) | (2-7) |
|  |  |  |  |
|  | Tanzania (1) | 24865±0 | 2±0 |
|  |  |  |  |
|  | Thailand (13) | 26281.8±945 | 3.3±0.7 |
|  |  | (25887-28932) | (3-5) |
|  |  |  |  |
|  | Uganda (3) | 26902±2196 | 3.7±1.7 |
|  |  | (24868-29951) | (2-6) |
|  |  |  |  |
|  | USA (8) | 27071±2008 | 3.9±1.6 |
|  |  | (24865-30007) | (2-6) |
| Mating type (Sample size) | MAT a(10) | 25265.5±692 | 2.3±0.5 |
|  |  | (24763-26941) | (2-3) |
|  |  |  |  |
|  | MAT alpha(154) | 26472±1804 | 3.4±1.5 |
|  |  | (24740-31327) | (2-7) |
|  |  |  |  |
|  | MAT alpha/alpha(12) | 26796.3±1843 | 3.4±1.4 |
|  |  | (24740-28932) | (2-5) |
|  |  |  |  |
|  | MATa/a(1) | 29002±0 | 7±0 |
|  |  |  |  |
|  | MATa/alpha(7) | 29002±0 | 7±0 |
| Source (Sample size) | Bird guano(7) | 25902.1±1355 | 3±1.3 |
|  |  | (24865-27974) | (2-5) |
|  |  |  |  |
|  | Cat(4) | 28932±0 | 5±0 |
|  |  |  |  |
|  | Clinical(139) | 26727.8±1900 | 3.6±1.7 |
|  |  | (24740-31327) | (2-7) |
|  |  |  |  |
|  | Environmental(31) | 25628.1±1039 | 2.7±0.9 |
|  |  | (24740-29154) | (2-5) |
|  |  |  |  |
|  | Unknown(3) | 24881±14.4 | 2±0 |
|  |  | (24868-24901) |  |
| Grand Total (184) |  | 25628.9±1825 | 3.5±1.6 |
|  |  | (24740-31327) | (2-7) |
